# Twist-diameter coupling drives DNA twist changes with salt and temperature

**DOI:** 10.1126/sciadv.abn1384

**Published:** 2022-03-23

**Authors:** Chen Zhang, Fujia Tian, Ying Lu, Bing Yuan, Zhi-Jie Tan, Xing-Hua Zhang, Liang Dai

**Affiliations:** 1 College of Life Sciences, The Institute for Advanced Studies, State Key Laboratory of Virology, Hubei Key Laboratory of Cell Homeostasis, Wuhan University, Wuhan 430072, China.; 2 Department of Physics, City University of Hong Kong, Hong Kong 999077, China.; 3 Institute of Physics, Chinese Academy of Sciences, Beijing 100190, China.; 4 Songshan Lake Materials Laboratory, Dongguan, Guangdong 523808, China.; 5 School of Physics and Technology, Wuhan University, Wuhan 430072, China.; 6 Shenzhen Research Institute, City University of Hong Kong, Shenzhen 518057, China.

## Abstract

DNA deformations upon environmental changes, e.g., salt and temperature, play crucial roles in many biological processes and material applications. Here, our magnetic tweezers experiments observed that the increase in NaCl, KCl, or RbCl concentration leads to substantial DNA overwinding. Our simulations and theoretical calculation quantitatively explain the salt-induced twist change through the mechanism: More salt enhances the screening of interstrand electrostatic repulsion and hence reduces DNA diameter, which is transduced to twist increase through twist-diameter coupling. We determined that the coupling constant is 4.5 ± 0.8 *k*_B_T/(degrees∙nm) for one base pair. The coupling comes from the restraint of the contour length of DNA backbone. On the basis of this coupling constant and diameter-dependent DNA conformational entropy, we predict the temperature dependence of DNA twist Δω_bp_/Δ*T* ≈ −0.01 degree/°C, which agrees with our and previous experimental results. Our analysis suggests that twist-diameter coupling is a common driving force for salt- and temperature-induced DNA twist changes.

## Teaser

DNA overwinds when adding salt or lowering temperature, which is mainly driven by DNA twist-diameter coupling.

## INTRODUCTION

Biological functions and material applications of DNA critically depend on the responses of DNA structure to many factors, such as force (*1*–*5*), temperature (*6*, *7*), and salt (*8*–*12*), because even subtle structural changes in DNA can lead to great effects in many cases, such as DNA-protein interactions (*13*–*15*) and DNA nanostructures from self-assembly (*16*, *17*). Such great effects are often caused by the sensitivity of relevant interactions or DNA organizations on DNA structures. For example, a small change in DNA twist per base pair can accumulate along long DNA molecules and leads to many turns of DNA rotation.

How DNA structure changes with force, temperature, or salt is not straightforward and often counterintuitive due to the sophisticated competition of various interactions in DNA. For example, people may expect that stretching DNA decreases its twist, but experiments observed the opposite trend (*1*, *18*). DNA deformations are often associated with the couplings among DNA structural parameters. For example, stretch-induced DNA twist change is caused by twist-stretch coupling (*1*, *19*–*21*). Again, because of the competition of various interactions, the molecular mechanism underlying twist-stretch coupling is not obvious and has been extensively investigated in many studies, including several recent ones (*1*, *18*, *20*–*22*). Similarly, researchers observed twist-bend coupling (*23*, *24*) in recent years. These couplings make substantial impacts on biological processes of DNA (*13*, *25*), such as DNA packaging in vivo (*23*).

While DNA deformation by force becomes clear after many years of effort (*1*, *18*, *20*, *21*), DNA deformations by salt and temperature are less understood, particularly in terms of the molecular mechanism underlying these DNA deformations. Here, we first report our experimental result of DNA twist change with salt. Then, using atomistic simulations and theoretical calculation, we reveal a strong twist-diameter coupling responsible for salt-induced DNA twist change. The molecular mechanism underlying this coupling is also unveiled. Very intriguingly, we find that this twist-diameter coupling can quantitatively explain temperature-induced DNA twist change observed in previous and our experiments, which suggests that twist-diameter coupling is a common driving force for salt- and temperature-induced DNA twist changes.

## RESULTS

### Salt-induced DNA twist change

**Fig. 1.**
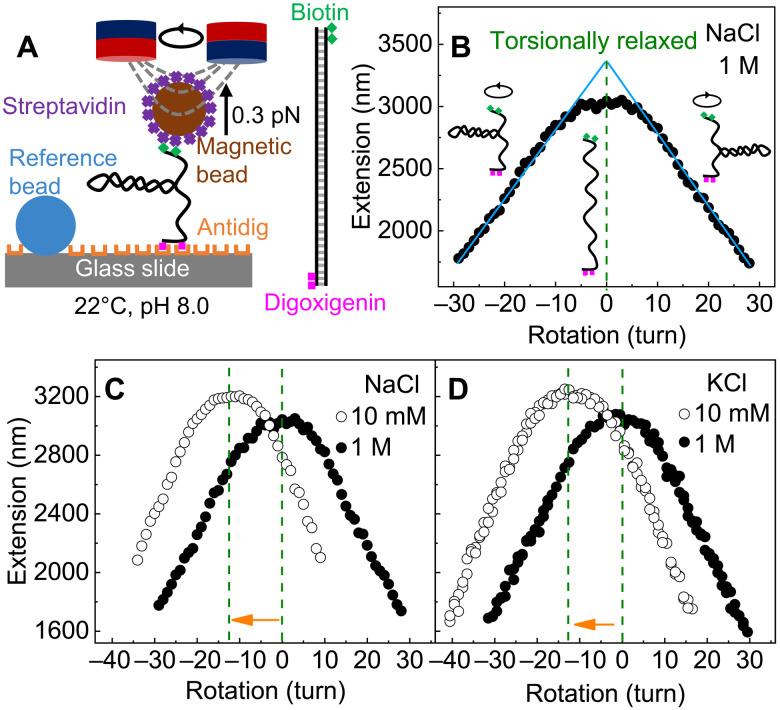
Measuring the changes in DNA twist caused by the increase in salt concentration by MT. (**A**) MT setup. One end of the DNA is labeled with multiple digoxigenin groups and anchored to a glass slide. The other end of the DNA is labeled with multiple biotin groups and attached to a superparamagnetic microbead. A pair of NdFeB magnets is used to rotate DNA at a constant force of 0.3 pN. (**B**) Determination of the torsionally relaxed point of DNA by the symmetrical centerline (green dashed line) of the rotation-extension curve. The two linear fits of the plectoneme range (cyan lines) meet at the torsionally relaxed point. (**C** and **D**) Determination of the change in DNA twist calculated using the shift in torsionally relaxed point caused by the decrease in salt concentration.

We first measured the DNA twist change with salt using magnetic tweezers (MT) (*6*, *8*), as illustrated in Fig. 1A. We performed all the experiments at 22°C, 10 mM tris-HCl (pH 8.0), and 0.3 pN for different salt concentrations (see more details in section S1). We rotated a single torsionally constrained double-stranded DNA by magnetic fields, measured the DNA extension simultaneously, and eventually obtained the rotation-extension curves as shown in Fig. 1B. For each rotation-extension curve, we determined the torsionally relaxed point of DNA (green dashed line) by the crossing point of the two linear fits of the plectoneme range (cyan solid lines in Fig. 1B). The number of rotation turns of the torsionally relaxed DNA is denoted as $N_{\text{turn}}^{*}$, which changes with the salt concentration (green dashed lines in Fig. 1, C and D). Accordingly, we calculated the equilibrium DNA twist angle per base pair, ω_exp_, from $N_{\text{turn}}^{*}$ through $\omega =(N_{\text{turn}}^{*}\times 360{}^{\circ})/N_{\text{bp}}$, where *N*_bp_ ≈ 13.6 × 10^3^ is the number of base pairs in the DNA. Because of the large *N*_bp_, we can determine Δω with the resolution on the order of 0.01° per base pair. Note that our experiment only gives the relative twist angle, i.e., DNA twist change induced by the variation of the salt concentration, *c*_salt_.

**Fig. 2.**
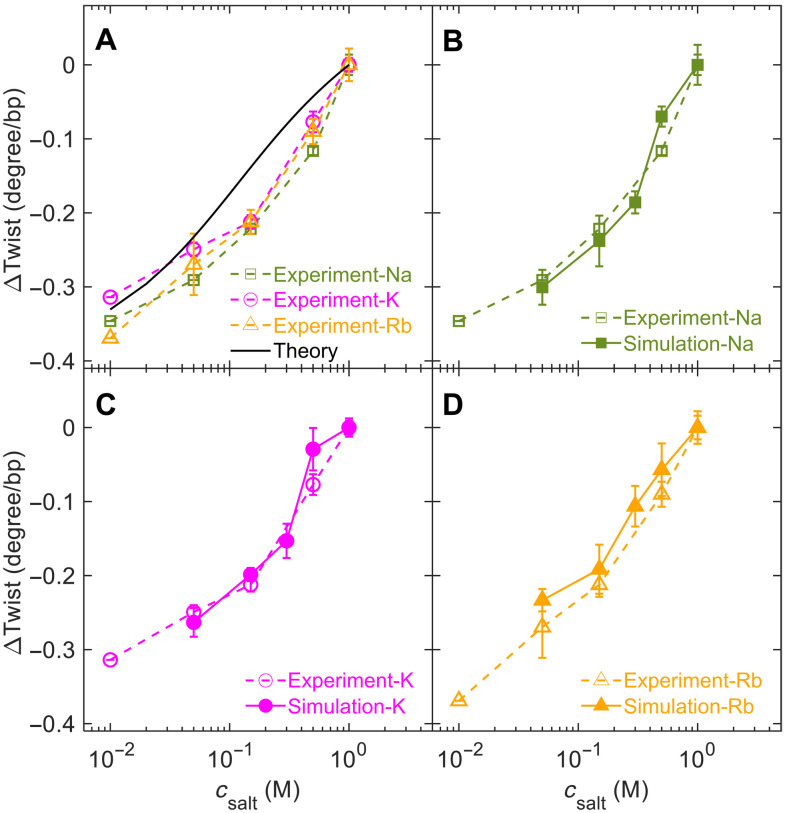
Twist angle as a function of the salt concentration determined by experiments, simulations, and theoretical calculations. The SDs obtained from more than three independent experiments or five simulation trajectories of 100 ns are shown in error bars. (**A**) Experimental results of the change in twist angle as a function of the salt concentration for NaCl, KCl, and RbCl. The black line is the theoretical result from Eq. 5. (**B** to **D**) Comparison of experimental results and simulation results for three salt species.

Figure 2A shows the experimental results of the relative DNA twist angle, Δω, as a function of *c*_salt_ for three salts: NaCl, KCl, and RbCl. We set Δω = 0 at *c*_salt_ = 1 M so that the data in Fig. 2A correspond to the change in DNA twist with respect to the value at 1 M. We find that Δω exhibits similar behavior for all three salts and agrees with the previous results based on DNA supercoiling measurements (*26*). These results suggest that the salt-induced twist change should be largely caused by the electrostatic screening effect of ions, while the subtle differences in the salt-induced twist changes among these three salts may be caused by the small differences in ion binding or ion distribution around DNA (*9*, *27*–*32*). The reason why we used the ions of Na^+^, K^+^, and Rb^+^ here is that these ions are likely to affect DNA structures mainly through electrostatic screening rather than specific binding.

### Reproduction of salt-induced twist change by simulations

**Fig. 3.**
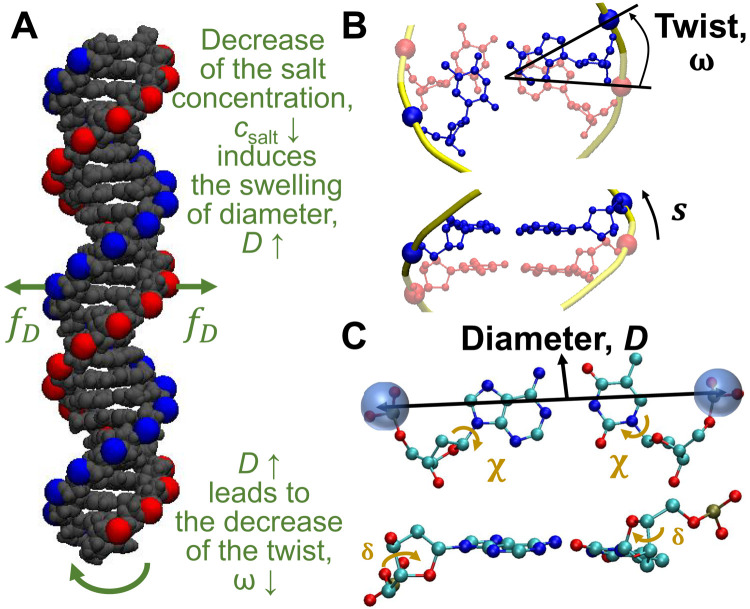
Mechanism underlying twist-diameter coupling. (**A**) Illustration of the salt-induced twist change. The decrease in *c*_salt_ induces the swelling of DNA diameter, which leads to the decrease in DNA twist through the negative twist-diameter coupling. The DNA structure is from our simulation snapshot. The red and blue beads represent negatively charged phosphate groups on two strands, which repel each other through electrostatic repulsions. (**B**) Top view and side view of 1-bp step to illustrate the twist angle, ω, and the backbone contour length, *s*, per base pair step. (**C**) Top view and side view of 1 bp to illustrate the DNA diameter in our calculation and two dihedral angles, χ and δ, mediating twist-diameter coupling.

To reveal the molecular mechanism for salt-induced twist change in our experiments, we performed all-atom molecular dynamics (MD) simulations of 25–base pair (bp) B-form DNA with the sequence (*33*) CGACTCTACGGAAGGGCATCTGCGC. The simulations were implemented in the GROMACS 2018.4 software (*34*) using OL15 force field (section S2) (*35*). Figure 3A presents one DNA structure from our simulation snapshot. For each concentration of NaCl, KCl, or RbCl, we performed 600-ns simulation and calculated DNA twist angles using the program Curves+ (*36*). As shown in Fig. 2 (B to D), our simulations quantitatively reproduced the experimental salt-induced twist changes. We did not simulate *c*_salt_ = 0.01 M because such low concentration requires a large simulation box, which is computationally too expensive.

### Twist change driven by twist-diameter coupling

Our analysis of MD simulations, together with theoretical calculations, suggests a mechanism for the salt-induced twist change: The decrease in *c*_salt_ induces the swelling of DNA diameter, which leads to the decrease in DNA twist through twist-diameter coupling, as illustrated in Fig. 3A. In the following part, we will present the evidence for this mechanism and the calculations of the twist-diameter coupling constant.

**Fig. 4.**
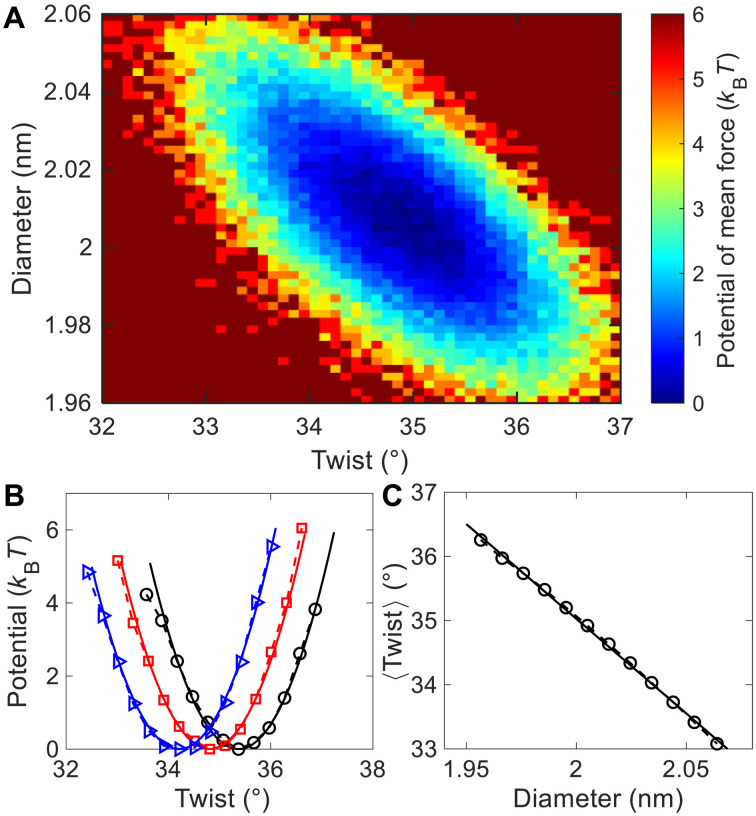
Free-energy analysis of the simulation result at 1 M NaCl. (**A**) Two-dimensional PMF with respect to DNA twist angle and DNA diameter calculated from the simulation. (**B**) One-dimensional PMF with respect to DNA twist angle for a narrow region of DNA diameter. The black, red, and blue symbols correspond to three regions of DNA diameter: 1.984 < *D* < 1.994 nm, 2.004 < *D* < 2.014 nm, and 2.024 < *D* < 2.034 nm, respectively. The solid lines are quadratic fits: *y* = 1.7(*x* − 35.37)^2^, *y* = 1.7(*x* − 34.81)^2^, and *y* = 1.7(*x* − 34.21)^2^, respectively. (**C**) Average twist angle as a function of the diameter. The symbols are from our simulation, and the solid line is a linear fit: *y* = −29.5*x* + 94.0.

Figure 4A shows a two-dimensional potential of mean force (PMF) as a function of the twist angle, ω, and the diameter, *D*. This PMF, *P*_sim_, was calculated from the simulation with 1 M NaCl through

$$P_{\text{sim}}(\omega ,D)=-(k_{B}T)\text{ln}[\Omega (\omega ,D)]$$ (1)

where *k*_B_ is the Boltzmann constant, *T* is the temperature, and Ω(ω, *D*) is the relative density of DNA conformations for a given ω and *D* from the simulation. We calculated Ω(ω, *D*) using the following procedure. Our MD simulations sampled *N*_conf_ = 5 × 10^4^ DNA conformations in equilibrium. For each DNA conformation, we computed ω*_i_* and *D_i_*, where *i* = 1,2,…, *N*_conf_ is the index of conformation. Then, these *N*_conf_ data points of {ω*_i_*, *D_i_*} were grouped into 10 × 10 bins according to the values of ω and *D*. For example, one bin corresponds to 34.5° < ω ≤ 35° and 1.95 nm < *D* ≤ 1.96 nm (see section S5). Next, the number of data points in each bin is counted and recorded as Ω(ω, *D*). When converting Ω(ω, *D*) to *P*_sim_(ω, *D*) using Eq. 1, we subtract a constant from *P*_sim_(ω, *D*) to make its minimum value to be zero. Here, we define the diameter *D* as the average distance between the two phosphate groups of 1 bp. The position of the phosphate group is defined as the center of mass of one P and two O atoms. It is worth to mention that the definition of DNA diameter has certain arbitrariness due to the irregular DNA surface structures. It should be reasonable to use interstrand phosphate distance for diameter considering that phosphate groups are outermost atoms. Furthermore, as will be shown later, such definition of DNA diameter can quantitatively reproduce DNA Young’s modulus.

The PMF at 1 M NaCl in Fig. 4A has a global minimum at ω_0_ ≈ 34.82° and *D*_0_ ≈ 2.008 nm. The two-dimensional PMF exhibits a valley extending from the left-top to the right-bottom, indicating a negative coupling between ω and *D*. Similar to the analyses of twist-stretch coupling in previous studies (*1*, *21*, *37*), we extracted the twist-diameter coupling constant by fitting the PMF using the following equation

$$P_{\text{bp}}\approx k_{\omega }^{\text{bp}}{\left(\Delta \omega \right)}^{2}/2+k_{D}^{\text{bp}}{\left(\Delta D\right)}^{2}/2+k_{\omega D}^{\text{bp}}\Delta \omega \Delta D$$ (2)

In the above equation, the cross term with $k_{\omega D}^{\text{bp}}\neq 0$ leads to a coupling between Δω and Δ*D*. The coupling can be understood in the following manner. For a given Δ*D*, the potential is minimized at a certain Δω^*^, and the value of Δω^*^ varies with Δ*D*. For example, in Fig. 4A, horizontal lines at different heights (different diameters) have different minimum locations (different twist values). By the fitting, we obtained the following coefficients for 1 M NaCl

$$k_{\omega }^{\text{bp}}\approx 0.18\pm 0.02\;k_{B}T/{\text{deg}}^{2}$$

$$k_{D}^{\text{bp}}\approx 263\pm 39\;k_{B}T/{\mathrm{nm}}^{2}$$

$$k_{\omega D}^{\text{bp}}\approx 4.5\pm 0.8\;k_{B}T/(\text{deg}∙\text{nm})$$ (3)

where Δω ≡ ω − ω_0_ and Δ*D* ≡ *D* − *D*_0_. The coefficients $k_{\omega }^{\text{bp}}$ and $k_{D}^{\text{bp}}$ characterize the rigidity of twist and diameter, respectively, while $k_{\omega D}^{\text{bp}}$ is the twist-diameter coupling constant. Note that these coefficients correspond to the relevant free energy per base pair as indicated by the superscript of “bp.” We determined $k_{\omega }^{\text{bp}}$, $k_{D}^{\text{bp}}$, and $k_{\omega D}^{\text{bp}}$ by the fit to the PMF in Fig. 4A using Eq. 2. The uncertainties in Eq. 3 correspond to 95% confidence interval during the fitting. We can convert $k_{D}^{\text{bp}}\approx 263\;k_{B}T/{\mathrm{nm}}^{2}$ to DNA Young’s modulus of 3.2 × 10^8^ Pa (see section S14) in agreement with the experimental value of 3.46 × 10^8^ Pa (*38*). As indicated by Eq. 2 and shown in Fig. 4B, for a given *D*, the PMF can be approximated by a harmonic potential, and the location of the potential minimum shifts toward a smaller ω with the increase in *D*. Figure 4C confirms that the dependence of average twist ⟨ω⟩ on *D* can be well captured by a straight line for a wide range of *D*.

Recall that the values of ω_0_, *D*_0_, $k_{\omega }^{\text{bp}}$, $k_{D}^{\text{bp}}$, and $k_{\omega D}^{\text{bp}}$ in Eq. 3 were calculated from the simulation with 1 M NaCl. We also calculated ω_0_, *D*_0_, $k_{\omega }^{\text{bp}}$, $k_{D}^{\text{bp}}$, and $k_{\omega D}^{\text{bp}}$ from the simulations with other *c*_salt_ and the salt of KCl and RbCl (see section S5). We find that $k_{\omega }^{\text{bp}}$, $k_{D}^{\text{bp}}$, and $k_{\omega D}^{\text{bp}}$ do not vary much for all three salt species of NaCl, KCl, and RbCl and all *c*_salt_ in our simulations from 0.05 to 1 M, which agrees with our theoretical prediction (see section S10). In all our simulations, *k*_ω*D*_ varies between 3.1 and 5.3 *k*_B_*T*/(deg∙nm). On the basis of the values of $k_{\omega }^{\text{bp}}$, $k_{D}^{\text{bp}}$, and $k_{\omega D}^{\text{bp}}$ in Eq. 2, we can determine the twist rigidity for a relaxed DNA, by ${\tilde{k}}_{\omega }^{\text{bp}}=k_{\omega }^{\text{bp}}-{\left(k_{\omega D}^{\text{bp}}\right)}^{2}/k_{D}^{\text{bp}}\approx$ 0.103 *k*_B_*T*/deg^2^, which corresponds to a twist rigidity of 470 pN∙nm^2^ and agrees with previous experimental (*19*, *39*–*41*) and simulation (*20*, *21*) results (see section S9).

### Reproduction of salt-induced twist change by theoretical calculation

After quantifying twist-diameter coupling by Eq. 2, we proceed to the theoretical calculations of how the twist-diameter coupling leads to salt-induced twist change. The basic idea of our calculation is as follows. The electrostatic repulsions between negatively charged P atoms on two DNA strands produce a force, *f_D_*, to increase DNA diameter. The formula of *f_D_* has been derived by Manning using the helical distribution of charges (*42*–*44*). It is expected that the decrease in *c*_salt_ increases *f_D_* and enlarges *D*, which eventually leads to the decrease in ω. To capture this salt-dependent twist change, we add a term −*f_D_D* into the PMF in Eq. 2. Note that in the term of salt-dependent twist change, we need the change of *f_D_* upon *c*_salt_ variation instead of the absolute value of *f_D_*. Accordingly, we define the relative Δ*f_D_* in line with the PMF in Eq. 2

$$\Delta f_{D}(c_{\text{salt}})\approx f_{D}(c_{\text{salt}})-f_{D}(1\;M)$$ (4)

Then, we can write the PMF as

$$P(c_{\text{salt}})\approx -\Delta f_{D}\Delta D+\frac{1}{2}k_{\omega }^{\text{bp}}{\left(\Delta \omega \right)}^{2}+\frac{1}{2}k_{D}^{\text{bp}}{\left(\Delta D\right)}^{2}+k_{\omega D}^{\text{bp}}\Delta \omega \Delta D$$ (5)

The term of −Δ*f_D_*Δ*D* is an approximation based on the fact that for a given *c*_salt_, Δ*f_D_* remains almost unchanged within the small *D* range we are dealing with. Minimizing *P*(*c*_salt_) with respect to Δω and Δ*D* yields

$$\Delta \omega (c_{\text{salt}})=\frac{-k_{\omega D}^{\text{bp}}}{k_{\omega }^{\text{bp}}k_{D}^{\text{bp}}-{\left(k_{\omega D}^{\text{bp}}\right)}^{2}}\Delta f_{D}$$ (6)

(see the minimization calculation in section S10). Substituting Δ*f_D_*(*c*_salt_) calculated from the Manning formula (see section S3) into Eq. 6, we obtained Δω(*c*_salt_) as shown by the black line in Fig. 2A, which agrees fairly well with the experimental and simulation results. The physical meaning of Eqs. 4 to 6 is as follows. Lowering the salt concentration enhances interstrand electrostatic repulsion in DNA, which produces an effective force, Δ*f_D_*, that swells DNA diameter. The force exerted on DNA diameter is eventually transduced to DNA twist through a total potential in Eq. 5.

To validate the above theoretical calculations, we performed two additional sets of simulations. In one set of simulations, we manually added external forces (equivalent to Δ*f_D_*) on P atoms of DNA and then measured the change in DNA twist. We obtained results in excellent agreement with Eq. 6 (see fig. S9). In the other set of simulations, we artificially modified the charge of each P atom from −1*e* to −1.5*e* or −0.5*e*. As expected, we observed a reduction of ω for the P charge of −1.5*e* and a rise of ω for the P charge of −0.5*e*, compared with the case of the P charge of −1*e* (see fig. S10). These results confirm that the salt-induced twist change is caused by the twist-diameter coupling and the variation of P-P electrostatic repulsions.

### The coupling mainly comes from DNA backbone stretch modulus

It is of interest to find out the molecular mechanism underlying twist-diameter coupling. One likely mechanism is that twist-diameter coupling is caused by the restraint of the contour length of DNA sugar phosphate backbone per base pair step, *s*, as illustrated in Fig. 3B. We can approximate $s\approx \sqrt{[{\left(\omega D/2\right)}^{2}+h^{2}}$, where *h* is the rise along the helical axis per base pair step. Because of the restraint of *s*, the increase in *D* leads to the decrease in ω. The stretch modulus of DNA backbone was estimated to be *S_h_* ≈ 965 pN by Gore *et al.* (*1*). The stretch modulus determines the energetic penalty when changing DNA sugar phosphate backbone length, for example, increasing both *D* and ω. Using a weak perturbation approximation for backbone length variation (see section S12), we can relate the stretch modulus to twist-diameter coupling constant, i.e., the prefactor of the cross term in Eq. 2, by the following equation

$${\left[k_{\omega D}^{\text{bp}}\right]}_{\text{est}}=\frac{S_{h}D_{0}^{3}\omega_{0}^{3}}{8s_{0}^{3}}\approx 2.8\;k_{B}T/(\text{deg}∙\text{nm})$$ (7)

where *D*_0_ ≈ 2.008 nm, ω_0_ ≈ 34.82° ≈ 0.6077 rad, and *s*_0_ ≈ 0.694 nm are the equilibrium values of the diameter, twist, and backbone length, respectively. Substituting the values of *S_h_*, *D*_0_, ω_0_, and *s*_0_, we very roughly estimate the twist-diameter coupling constant as 2.8 *k*_B_T/(deg∙nm), which agrees fairly well with the value in Eq. 3 considering that we neglect many factors (see section S12).

### The coupling is mediated by two dihedral angles

We analyzed the structural basis of twist-diameter coupling and found that the coupling is mediated through two dihedral angles in DNA backbone as illustrated in Fig. 3C. These two dihedral angles are commonly referred to as χ and δ. We found that both χ and δ are strongly and negatively correlated with the diameter while strongly and positively correlated with the twist. The increase in *D* under a lower salt concentration is realized through the decrease in χ and δ, which simultaneously reduces ω (see section S4).

### The coupling also drives temperature-induced DNA twist change

**Fig. 5.**
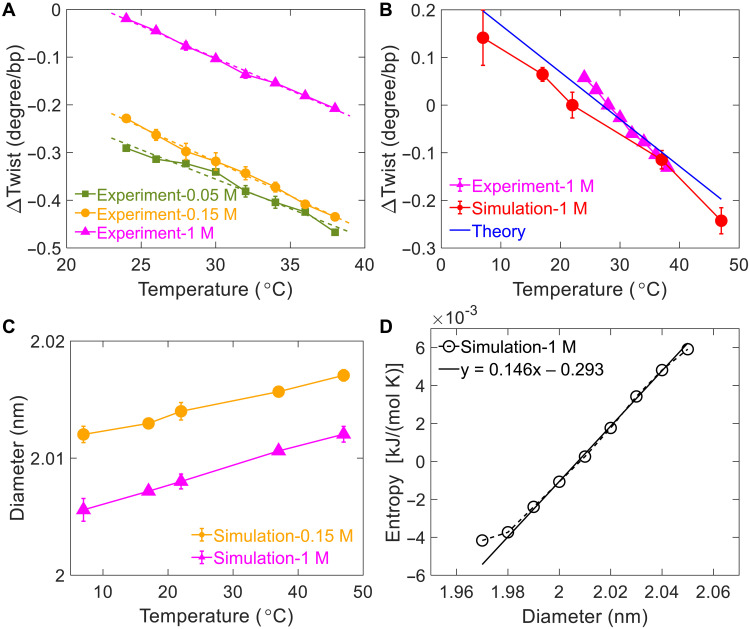
Temperature-induced DNA twist change. (**A**) MT experimental results of DNA twist change with temperature for 0.05, 0.15, and 1 M NaCl. ΔTwist is set to zero at 22°C and 1 M NaCl. Linear fits to experimental data yield the slopes of −0.0123 ± 0.002 deg/°C for 0.05 M, −0.0144 ± 0.0009 deg/°C for 0.15 M, and −0.0135 ± 0.0007 deg/°C for 1 M NaCl. (**B**) Comparison of results from experiments, MD simulations, and theoretical prediction from Eq. 10. (**C**) Dependence of average DNA diameter on temperature from MD simulations. (**D**) Dependence of DNA conformational entropy on diameter extracted from MD simulations at 1 M NaCl.

Our analysis suggests that twist-diameter coupling also drives temperature-induced DNA twist change. We measured DNA twist change with temperature just like the case of the salt effect as shown in Fig. 5A. Our experimental results agree with a recent experimental study (*6*). The experimental results were reproduced by our simulation and theoretical calculation (Fig. 5B). The basic idea of the theoretical calculation is as follows. The competition of interstrand attraction *U*(*D*) and DNA conformational entropy *S*(*D*) leads to an equilibrium DNA diameter at the minimum of free energy *F_T_* = *U* − *TS*. Obviously, DNA conformational entropy increases with the diameter because a large diameter allows more freedom for the relative motion of two strands. A higher temperature enhances the weight of entropy in the free energy *F_T_* = *U* − *TS*, which leads to diameter swelling, just like thermal expansion in most materials. The change in temperature, Δ*T*, exerts an effective force, Δ*f_T_*, on DNA diameter

$$\Delta f_{T}=\Delta T\frac{\partial S}{\partial D}\approx k_{\text{SD}}\times \Delta T\;\text{with}\;k_{\text{SD}}\equiv \frac{\partial S}{\partial D}\approx 0.146\;\text{kJ}/(\text{mol}\;K\;\text{nm})$$ (8)

Here, *k*_SD_ characterizes diameter dependence of entropy and was calculated by simulation results in Fig. 5D. The calculation detail of *k*_SD_ can be found in section S11. The physical reason for the diameter dependence of entropy is simple: A large diameter corresponds to more conformational space. Similar to Eq. 6, the effective force Δ*f_T_* on DNA diameter is transduced to the change in the twist through twist-diameter coupling

$$\Delta \omega_{\text{bp}}(\Delta T)=\frac{-k_{\omega D}^{\text{bp}}}{k_{\omega }^{\text{bp}}k_{D}^{\text{bp}}-{\left(k_{\omega D}^{\text{bp}}\right)}^{2}}\Delta f_{T}=\frac{-k_{\omega D}^{\text{bp}}}{k_{\omega }^{\text{bp}}k_{D}^{\text{bp}}-{\left(k_{\omega D}^{\text{bp}}\right)}^{2}}k_{\text{SD}}\Delta T$$ (9)

Then, temperature-dependent twist change has a coefficient *k_T_*

$$k_{T}^{\text{bp}}\equiv \frac{\Delta \omega_{\text{bp}}}{\Delta T}=\frac{-k_{\omega D}^{\text{bp}}}{k_{\omega }^{\text{bp}}k_{D}^{\text{bp}}-{\left(k_{\omega D}^{\text{bp}}\right)}^{2}}k_{\text{SD}}$$ (10)

Substituting the parameters and using 1 *k*_B_*T* ≈ 2.454 kJ/mol at *T* = 22°C, we predict the coefficient

$$k_{T}^{\text{bp}}\approx -0.01\;\text{deg}/{}^{\circ}C$$ (11)

The above coefficient agrees with our experimental result in Fig. 5A, −0.0135 deg/°C, and a previous experimental result, −0.0110 deg/°C (*6*).

**Fig. 6.**
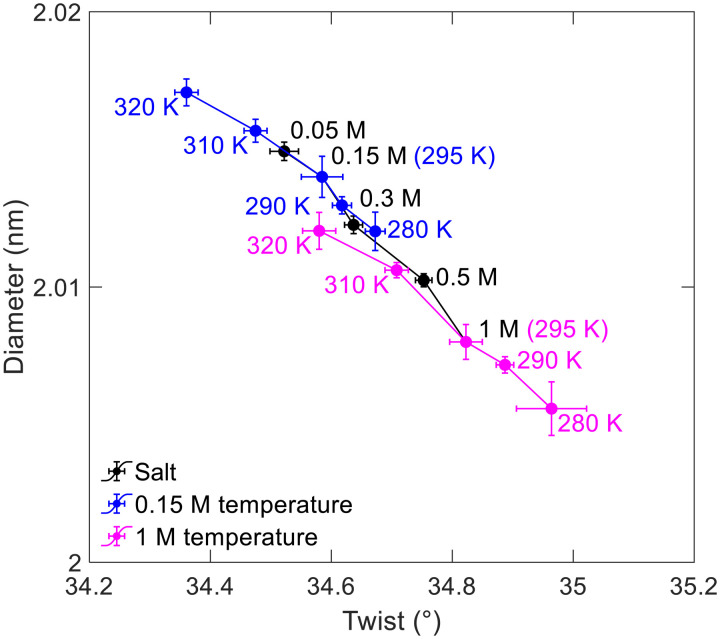
Collapse of the twist-diameter curves for salt and temperature effects. These three curves are calculated from the simulations that deform DNA by salt and temperature. Each data point corresponds to the average twist and the average diameter in one simulation at a given *c*_salt_ (fixing *T* = 295 K) or at a given temperature (fixing *c*_salt_ = 0.15 or 1 M).

To further support that twist-diameter coupling is the common driving force for salt- and temperature-induced twist changes, Fig. 6 compares the twist-diameter curves calculated from the simulations that deform DNA by salt and temperature. Each data point corresponds to the average twist and the average diameter in one simulation with fixed *c*_salt_ and *T*. These curves substantially collapse, which supports that salt- and temperature-induced twist changes are driven by the same factor: twist-diameter coupling.

In addition to salt- and temperature-induced twist changes, stretch-induced twist change is likely to be related to twist-diameter coupling as well (*1*, *18*, *20*, *21*). One mechanism proposed for stretch-induced twist change by Gore *et al.* (*1*) consists of two steps: (i) Stretching DNA causes the shrinking of DNA diameter due to DNA volume conservation; (ii) the shrinking of DNA diameter is transduced to DNA overwinding. The second step implies the twist-diameter coupling, i.e., the transduction from diameter change to twist change.

### Salt- and temperature-induced twist changes are mainly mediated by diameter variation and twist-diameter coupling

Varying salt concentration or temperature should affect many DNA structural parameters, not only DNA diameter but also others, including the contour length *L* and persistence length *L_p_* (*8*, *9*). Our analysis shows that the variation of the contour length upon salt change only plays a minor role in salt-induced twist changes (see section S6). The effect of salt on DNA twist should be mainly mediated by diameter variation rather than variations of other structural parameters for the following reasons. First, the salt-induced twist changes in experiments were quantitatively reproduced by salt-induced diameter variation and twist-diameter coupling as shown in Fig. 2A. Second, adjusting diameter in simulations by external forces quantitatively reproduced twist change (fig. S9). Third, theoretical calculation of electrostatic interaction quantitatively reproduced salt-induced diameter variation. Fourth, twist-diameter coupling constant obtained from MD simulation agrees fairly well with the one estimated the stretch modulus of DNA backbone, which suggests twist variation is directly linked to diameter variation rather than through a hidden structural parameter. Lastly, experimental temperature-induced twist change was quantitatively reproduced by diameter-dependent DNA conformational entropy and twist-diameter coupling. See section S13 for more discussions.

From the physical point of view, it is quite reasonable that DNA diameter plays a crucial role in mediating salt- and temperature-induced DNA deformations. Diameter quantifies phosphate-phosphate distance, which adjusts charge interactions in salt effects, and quantifies interstrand distance, which determines DNA conformational entropy in temperature effects.

### Confirmation of the salt effect and temperature effect by different DNA constructs in experiments

To examine whether the observed salt and temperature effects depend on the specific DNA construct (13 kbp with guanine-cytosine (GC) content 43%) used in our experiments, we have conducted the experiments for two additional DNA constructs (20 kbp with GC content 55% and 6 kbp with GC content 57%). While these DNA constructs differ significantly in DNA length, the experimental results of these three DNA constructs agree with each other (see fig. S19), which suggests that the salt- and temperature-induced DNA twist changes are not caused by the specific DNA construct. The insensitivity of the temperature-induced twist changes on the DNA construct has also been observed in the previous experiments by Kriegel *et al.* (*6*).

### Dependences of the twist-diameter coupling constant on the force field in simulations

To examine whether the simulation results depend on the force field, we have performed additional simulations using the Parmbsc1 force field, another popular DNA force field developed in recent years. The twist-diameter coupling constant per base pair at 1 M NaCl is 4.92 ± 0.92 *k*_B_T/(deg∙nm) for the Parmbsc1 force field (*45*) and 4.50 ± 0.82 *k*_B_T/(deg∙nm) for the OL15 force field (*35*). The differences are comparable with statistical errors.

## DISCUSSION

In this work, we find that salt- and temperature-induced DNA twist changes are driven by twist-diameter coupling. For salt effects, lowering the salt concentration enhances interstrand electrostatic repulsion and increases diameter, which is transduced to DNA underwinding through twist-diameter coupling. For temperature effects, a higher temperature causes DNA diameter swelling, which is transduced to DNA underwinding through twist-diameter coupling. We determined the twist-diameter coupling constant as approximately 4.5 ± 0.8 *k*_B_*T*/(deg∙nm) for 1 bp based on which we quantitatively reproduced experimental salt- and temperature-induced DNA twist changes.

Salt- and temperature-induced DNA twist changes should have significant implications for relevant biological processes (*23*) and material applications of DNA origami (*16*, *17*). Twist changes accumulate along DNA and can reach up to thousands of turns or more, depending on the DNA length. For 13.6-kbp DNA, the twist change can cause DNA rotation of several turns (Fig. 1). In cells, the twist change may cause thousands to millions of rotations for mega to giga base pair long.

## MATERIALS AND METHODS

### MT experiments

We built the MT and performed the experiments following the detailed instructions published previously (*6*, *46*, *47*). Briefly, we rotated the magnets one turn by one turn at a constant force of 0.3 pN. After each rotation turn, we recorded the extension in DNA for 10 s and calculated the average in extension. Usually, we measured the extension in DNA in the range of ±20 turns flanking the torsional relaxed point of the DNA, generating a bell-like torsion-extension curve. Then, we changed to another salt concentration (or another temperature) and measured the torsion-extension curve using the same DNA molecule. At each salt concentration (or each temperature), we used at least three DNA molecules in different flow cells to calculate the twist as a function of salt concentration (or temperature) (see more details in section S1).

### Atomistic MD simulations

All-atom MD simulations were performed with the GROMACS 2018.4 software package (*48*) and OL15 force field (*35*). DNA structural parameters were calculated using the program Curves+ (see more details in section S2) (*36*).

## Supplementary Materials

### This PDF file includes:

Sections S1 to S15 Figs. S1 to S19 Tables S1 to S4 References

## Notes

View/request a protocol for this paper from *Bio-protocol*.
