# Supplementary material for: Twist-diameter coupling drives DNA twist changes with salt and temperature
Source: Sci Adv. 2022 Mar 23;8(12):eabn1384. doi: 10.1126/sciadv.abn1384 (PMC8942373; doi:10.1126/sciadv.abn1384)
Supplement: Supplementary file 1 — Sections S1 to S15 Figs. S1 to S19 Tables S1 to S4 References [file sciadv.abn1384_sm.pdf]

Science Advances
AAAS

Supplementary Materials for

Twist-diameter coupling drives DNA twist changes with salt and temperature

Chen Zhang, Fujia Tian, Ying Lu, Bing Yuan, Zhi-Jie Tan, Xing-Hua Zhang*, Liang Dai*

*Corresponding author. Email: liangdai@cityu.edu.hk (L.D.); zhxh@whu.edu.cn (X.-H.Z.)

Published 23 March 2022, *Sci. Adv.* **8**, eabn1384 (2022)
DOI: 10.1126/sciadv.abn1384

This PDF file includes:

Sections S1 to S15
Figs. S1 to S19
Tables S1 to S4
References

Section S1. Details of single-DNA magnetic-tweezers experiments

(i) Temperature and solutions

We performed all single-molecule experiments in a cell culture room with a constant temperature of 22 ℃. We prepared filtrated, autoclaved stocks of 4 M NaCl, KCl, and RbCl (Sigma-Aldrich). We purchased 1 M Tris-HCl pH 8.0 buffer from Sigma-Aldrich. Just before single-molecule experiments, we diluted the salt and Tris-HCl stocks in autoclaved DI water to specific salt concentrations.

(ii) Torsionally constrained DNA

We prepared the torsionally constrained DNA using a megaprimer-PCR approach invented by Paik et al. (*49*). In the main text, we used the DNA sequences from 19116-31647 bp of lambda DNA containing 43.3% GC content, the same as the DNA used in our recent work (*8*). Briefly, we firstly amplified the two megaprimers either containing multiple biotin groups or digoxigenin groups by PCR with 30% biotin-11-dUTP or digoxigenin-11-dUTP. Then, we amplified the final DNA product using 19116-31647 bp of lambda DNA as the template together with the two megaprimers. We purified the final DNA product by agarose gel electrophoresis. In practice, we found ~70% DNA molecules were successfully torsion-constrained.

We also prepared 6 kbp and 20 kbp torsionally constrained DNA constructs following similar protocols. (1) We made a multiple-biotin-labeled short DNA by PCR using 6K_F:6K_Rs or 20K_F: 20K_Rs as primers together with 30% biotin-11-dUTP (Thermo Fisher Scientific) and using lambda DNA as the template. (2) We made a multiple-digoxigenin-labeled short DNA fragment by PCR using 6K_Fs:6K_R or 20K_Fs:20K_R as primers together with 30% digoxigenin-11-dUTP (Roche) and using lambda DNA as the template. (3) We made the torsionally constrained DNA constructs by PCR using the two labeled short DNA fragments as megaprimers and lambda DNA as the template.

The sequences of the oligos are listed.
20K_F: ATGACTGCTGCTGCATTGACG (3197-3217 of lambada DNA)
20K_R: CTCCGGCACATAGCAGTCCTAG (24344-24323 of lambada DNA)
20K_Fs: GCGAGAATTTTTAGCCCAAGC (23670-23690 of lambada DNA)
20K_Rs: GTAGCTCATCTGGGCGTAATTC (3981-3960 of lambada DNA)
6K_F: CCCACGCTGACGGTTTCTAACC (13657-13678 of lambada DNA)
6K_R: GTTTACCCGCAAGCGCGTTAG (21053-21033 of lambada DNA)
6K_Fs: CATCATCAAGTGCCGGTCGTGCAG (20407-20430 of lambada DNA)
6K_Rs: CGCTGATTCTGTCTGTGTCATG (14153-14132 of lambada DNA).

(iii) Flow cell

Briefly, we functionalized the Piranha-cleaned cover glass slides by 1% APTES (Sigma-Aldrich) - 2% glutaraldehyde (Sigma-Aldrich) - 0.1 mg/mL anti-digoxigenin (Roche), and

passivated them using 200 mM Tris-HCl pH 8.0 (Sigma-Aldrich) and 2% BSA (Sigma-Aldrich). We pasted two double-sided adhesive tapes to the functionalized cover glass slide and then put a smaller cover glass slide on the top to form a flow cell. The flow cell has a capacity of ~ 40 μL solution.

(iv) DNA and bead tethering

We diluted the DNA to ~ 2 ng/mL in 10 mM Tris-HCl, 500 mM NaCl and anchor the DNA to the glass slide of the flow-cell (~ 40 μL capacity) by 10 minutes incubation. Then, we diluted the microbeads (M-270 streptavidin, Dynal) to 100-fold and attached the beads to the free end of DNA by 10 minutes of incubation in the flow cell. The unbound beads are removed by excessively rinsing using about 10 mL 1 mM Tris-HCl pH 8.0.

(v) Torsion-extension curves measured by magnetic-tweezers

We built the magnetic-tweezers and performed the experiments following the detailed instructions published previously (*6, 46, 47*). Briefly, we rotated the magnets one turn by one turn at a constant force of 0.3 pN. After each rotation turn, we recorded the extension in DNA for ten seconds and calculated the average in extension. Usually, we measured the extension in DNA in the range of ±20 turns flanking the torsional relaxed point of the DNA, generating a bell-like torsion-extension curve. Then, we changed to another salt concentration and measured the torsion-extension curve using the same DNA molecule. At each salt concentration, we used at least three DNA molecules in different flow cells to calculate the twist as a function of salt concentration.

(vi) The contribution of 10 mM Tris-HCl buffer to the ionic strength.

The buffer used in our experiments is 10 mM Tris-HCl pH 8.0, which is a typical buffer in DNA experiments to stabilize the pH value of the solution. To determine the contribution of 10 mM Tris-HCl to the ionic strength, we measured the conductivity as a function of the concentration of NaCl or Tris-HCl pH 8.0 in the range of 0.1 to 100 mM, as shown in **Figure S1**. We found that the conductivity increases linearly with the salt concentration. The contribution of 10 mM Tris-HCl to the ionic strength is equivalent to about 5.3 mM NaCl at 22 ℃, as indicated by the two magenta crossing lines.

The DNA solutions in our experiments always include 10 mM Tris-HCl pH 8.0 plus the desired salt concentrations. Because the contribution of 10 mM Tris-HCl buffer to the ionic strength is much smaller than the added salt, we do not consider this contribution for simplicity. Recall that the minimum salt concentration is used in our simulations is 50 mM. Including the contribution of 10 mM Tris-HCl pH 8.0 to the ionic strength would very slightly affect the comparison of experimental and simulation results.

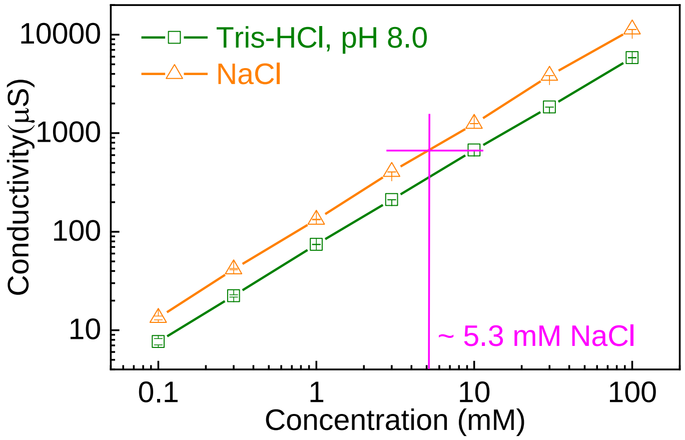

Fig. S1. The conductivity of Tris-HCl pH 8.0 and NaCl solutions at 22 ℃. The averages and standard deviations of the conductivity obtained from three measurements are shown as data points and error bars.

Section S2. Details of all-atom molecular dynamics simulations

The initial structure of 25-bp double-stranded DNA (dsDNA) with the sequence of CGACT CTACG GAAGG GCATC TGCGC (*33*) was built by the program 3DNA (*50*) in B-form. The DNA molecule was neutralized with counterions (Na^+, K^+ or Rb^+) and then immersed in a simulation box of 8×8×12.5 nm^3, which was filled with TIP3P water molecules. A certain number of salt ions were added to produce the desired salt concentrations (see a simulation snapshot in **Figure S2**). The interactions of ions were described by the force-field parameters developed by Joung and Cheatham (*51*). Each simulation started with energy minimization for 10000 steps, followed by thermalization in the canonical (NVT) ensemble and equilibrated in the isobaric-isothermal (NPT) ensemble with positional restraints on dsDNA for 10 ns. After the equilibration, the restraints were removed and a production run of 600 ns was carried out for every salt concentration of NaCl, KCl or RbCl. The last 500 ns of simulation was used for data analysis.

All-atom MD simulations were performed with the GROMACS 2018.4 software package (*48*) and OL15 force field (*35*). Periodic boundary conditions were used in all three dimensions. Particle Mesh Ewald method (*52*) was used for long-range electrostatic interactions. A 1.0 nm cut-off was applied for van der Waals interactions and short-range electrostatic interactions. The LINCS algorithm (*53*) was applied, and a 2 fs time step was used with the leap-frog integrator (*54*) for all simulations. The temperature of the system was coupled to 295 K using V-rescale thermostat (*55*) with a relaxation time τ = 0.1 ps and pressure was kept at 1 atm using Parrinello-Rahman pressure-coupling (*56*) with a relaxation time τ = 2.0 ps and compressibility 4.5×10^{-5} bar^{-1}. The coordinates were saved every 10 ps for MD trajectories.

DNA structural parameters were calculated using the program Curves+ (*36*). To avoid possible end effects, we only calculated the structural parameters for the middle 18 base pairs of the 25-bp DNA.

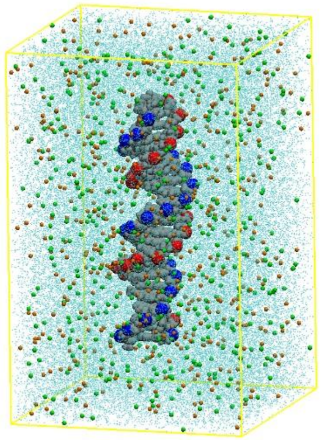

Fig. S2. A snapshot from the simulation with 1 M NaCl. Na^+ and Cl^- are represented by small orange and green beads, respectively. Water molecules are represented by very small blue dots.

Section S3. Theoretical calculations of f_D based on Manning's formula

The electrostatic free energy of charges on DNA has been derived by Manning based on counterion condensation theory (*42*, *43*). Manning has derived the electrostatic free energy for the single-helix model (*42*) and the double-helix model (*43*). Here, we employed the double-helix model to calculate the electrostatic free energy per base, P_{ES}^{base}:

$$P_{ES}^{base} = \frac{1}{Z}\left(2 - \frac{b_2}{Zl_B}\right)[STRUCTURE - \ln(1 - \exp(-\kappa b_1))] - \frac{1}{Z} + \frac{b_2}{Z^2 l_B} \qquad (S1)$$

with

$$STRUCTURE = S_0 + S_1 + S_+ + S_- \qquad (S2)$$

$$S_0 = \frac{1}{2}\left(\frac{b_2}{r_{00'}}\right)e^{-\kappa r_{00'}} \qquad (S3)$$

$$S_1 = \frac{1}{2}\sum_{n=1}^{\infty}\frac{1}{n}\left[\frac{e^{-\kappa r_n}}{\left(\frac{r_n}{nb_1}\right)} - e^{-n\kappa b_1}\right] \qquad (S4)$$

$$S_+ = \frac{1}{4}\sum_{n=1}^{\infty}\frac{1}{n}\left[\frac{e^{-\kappa r_{0n'}}}{\left(\frac{r_{0n'}}{nb_1}\right)} - e^{-n\kappa b_1}\right] \qquad (S5)$$

$$S_- = \frac{1}{4}\sum_{n=1}^{\infty}\frac{1}{n}\left[\frac{e^{-\kappa r_{0,-n'}}}{\left(\frac{r_{0,-n'}}{nb_1}\right)} - e^{-n\kappa b_1}\right] \qquad (S6)$$

In the $STRUCTURE$, $r_{00'}$ is the length of the chord from a charged site on the unprimed helix to the site 'directly across' from it on the primed helix. It is obtained by setting $n = 0$ in either Eq (S8) or (S9) presented below. r_n is the chord-distance between two charge sites on the same helix separated by $n - 1$ other sites on that helix, and can be found from Eq (S7). $r_{0n'}$ and $r_{0-n'}$ are interhelical chord-distances from Eq (S8) and Eq (S9), respectively.

$$r_n = nb_2\left\{1 + \left(\frac{2R^2}{n^2 {b_2}^2}\right)\left[1 - \cos\left(\frac{n\omega}{2}\right)\right]\right\}^{\frac{1}{2}} \qquad (S7)$$

$$r_{0n'} = nb_1\left\{\left(1 + \frac{\Delta\delta}{nb_1}\right)^2 + \frac{2R^2}{n^2 b_1^2}[1 - \cos(n\omega + \Delta\alpha)]\right\}^{\frac{1}{2}} \qquad (S8)$$

$$r_{0,-n'} = nb_1\left\{\left(1-\frac{\Delta\delta}{nb_1}\right)^2 + \frac{2R^2}{n^2b_1^2}[1-\cos(n\omega-\Delta\alpha)]\right\}^{\frac{1}{2}} \quad (S9)$$

and

$$l_B = \frac{e^2}{4\pi\epsilon_0\epsilon k_B T} \quad (S10)$$

$$\kappa = \sqrt{8\pi l_B N_A \times 10^3 c_{salt}} \quad (S11)$$

where $Z = 1$ is the valence of the counterion, $b_2 = b_1/2 = h/2 \approx 0.17\ nm$ is the half rise in the unit of nm, l_B is the Bjerrum length of water, e is the unit charge, ϵ_0 is the vacuum permittivity, $\epsilon_r \approx 78.4$ is the dielectric constant of water, k_B is the Boltzmann constant, $T = 295K$ is the temperature, κ is the inverse of the Debye screening length, $N_A \approx 6.022 \times 10^{23}$ is the Avogadro number, c_{salt} is the concentration of monovalent salt in M, $R = D/2$ is the DNA radius, and ω is the DNA twist angle. Note that in this double-helix model, the two helices are represented by

$$r(\phi) = [R\cos(\phi+\alpha), R\sin(\phi+\alpha), h\phi+\delta] \quad (S12)$$

$$r'^{(\phi)} = \left[R\cos\left(\phi+\alpha'\right), R\sin\left(\phi+\alpha'\right), h\phi+\delta'\right]. \quad (S13)$$

These two helices share the same radius R, the same rise h, and the same rotation angle ϕ, but with different phase angles α and α', and different vertical displacements δ and δ'. Then, we define $\Delta\alpha \equiv \alpha - \alpha'$ and $\Delta\delta = \delta - \delta'$, which are used in Eqs (S7-S9).

To be consistent with the main text, we define the electrostatic free energy per base pair, P_{ES}^{bp}, as:

$$P_{ES}^{bp} \equiv 2P_{ES}^{base}. \quad (S14)$$

Then, we calculated the effectively electrostatic force that tends to increase D:

$$f_D = -\left(\frac{\partial P_{ES}^{bp}}{\partial D}\right)_{\omega=\omega_0}. \quad (S15)$$

The numerical results of P_{ES}^{bp} and f_D in Eq (S1-S15) are plotted in **Figure S3**.

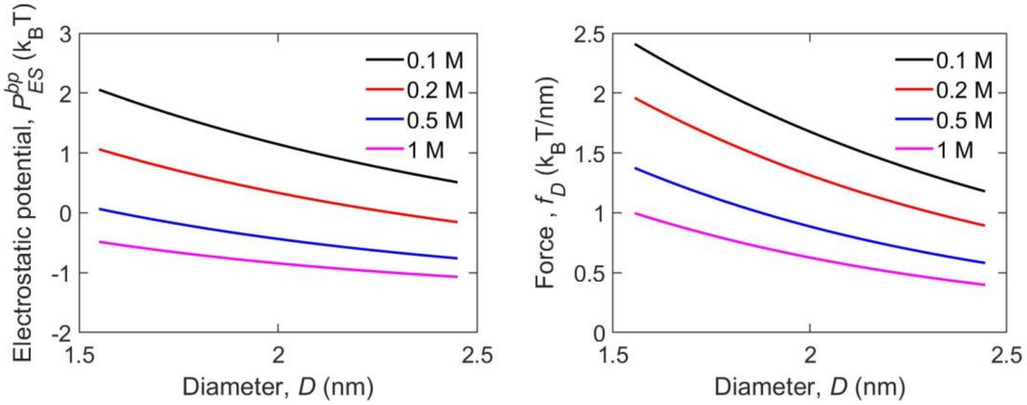

Fig. S3. **Theoretical results of electrostatic free energy and force.** Electrostatic free energy (left plot) and force (right plot) per base pair as a function of DNA diameter for the twist angle $\omega_0 = 34.82°$.

Section S4. Dihedral angles mediating the twist-diameter coupling

Figure S4 illustrates seven main dihedral angles in DNA structure. **Table S1** lists the correlation among these dihedral angles, DNA twist and diameter. We identified that the dihedral angles χ and δ have the strongest correlations with DNA twist and diameter.

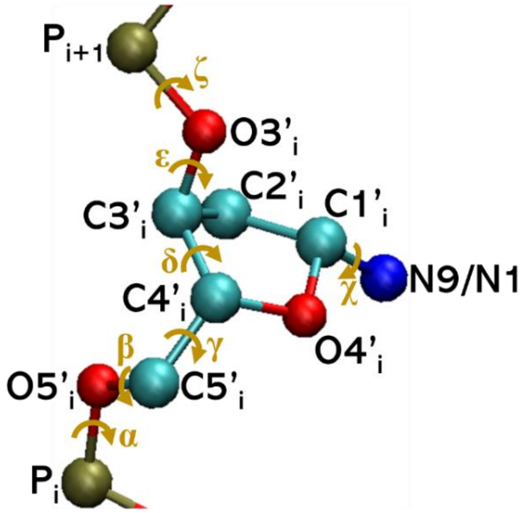

Fig. S4. **Illustration of seven dihedral angles on DNA structure.**

Table S1. Pearson correlation coefficients among the seven dihedral angles of DNA backbones, the twist angle, and the diameter from our MD simulation with 1 M NaCl. Note that the data in the top-right triangle are identical to the data in the bottom-left triangle. The coefficients with absolute values greater than 0.4 are marked in red.

	α	β	γ	δ	ε	ζ	χ	Twist	Diameter
α	1.0000	-0.0338	-0.4259	-0.0894	0.0327	-0.1410	-0.1062	0.0108	0.0858
β	-0.0338	1.0000	0.0781	-0.0684	-0.4694	0.2027	-0.0257	0.0743	-0.0772
γ	-0.4259	0.0781	1.0000	-0.2346	-0.0139	-0.1880	-0.1711	0.0624	-0.0328
δ	-0.0894	-0.0684	-0.2346	1.0000	-0.0817	0.1654	0.6336	0.4950	-0.5805
ε	0.0327	-0.4694	-0.0139	-0.0817	1.0000	-0.1243	0.0444	0.0038	0.0638
ζ	-0.1410	0.2027	-0.1880	0.1654	-0.1243	1.0000	0.3189	0.0528	-0.1849
χ	-0.1062	-0.0257	-0.1711	0.6336	0.0444	0.3189	1.0000	0.4314	-0.6365
Twist	0.0108	0.0743	0.0624	0.4950	0.0038	0.0528	0.4314	1.0000	-0.6922
Diameter	0.0858	-0.0772	-0.0328	-0.5805	0.0638	-0.1849	-0.6365	-0.6922	1.0000

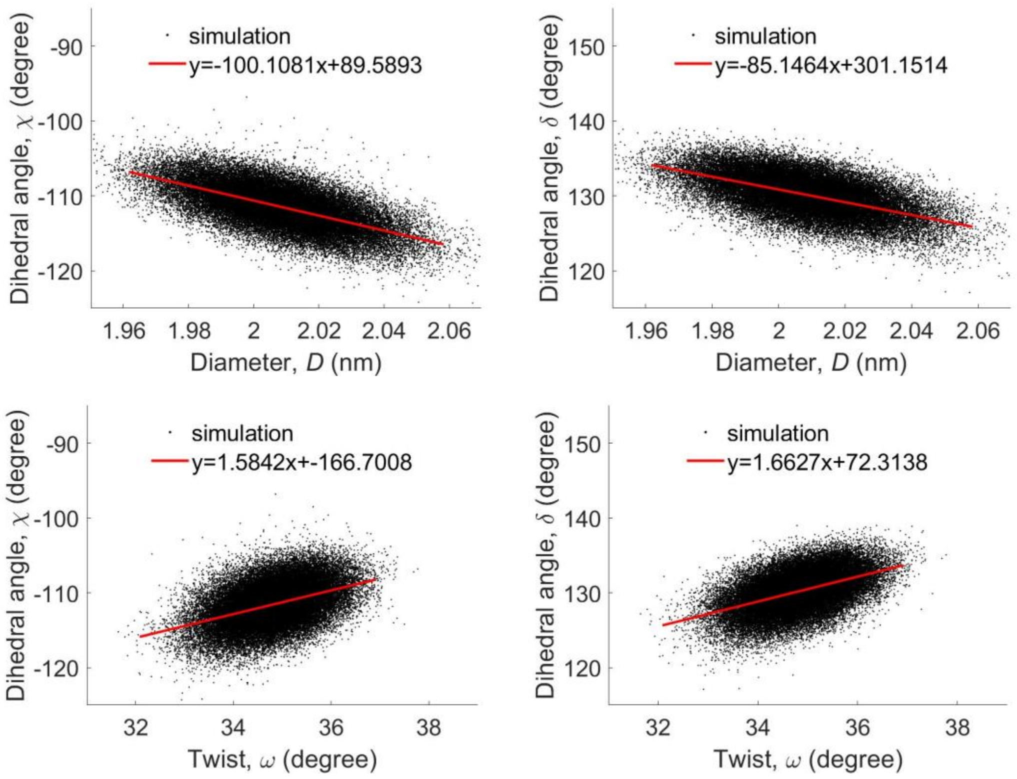

Fig. S5. **Correlations among DNA structural parameters.** Scatter plots for twist or diameter versus the dihedral angle χ or δ from the simulation at 1 M NaCl. Each dot corresponds to the data from one DNA conformation in the simulation.

Section S5. Twist-diameter coupling constants at different salt concentrations

Table S2 displays the PMF obtained from the simulation with 1 M NaCl. The data in Table S2 were used to determine k_{ω}^{bp}, k_{D}^{bp} and $k_{\omega D}^{\text{bp}}$ in Eq (2) through fitting.

Table S3 and Figure S6 present k_{ω}^{bp}, k_{D}^{bp}, $k_{\omega D}^{\text{bp}}$, ω_0, and D_0 obtained by the fits to the simulation PMFs with different salt concentrations and salt species.

Table S2. The two-dimensional potential of mean force (PMF) in 10×10 bins with respect to DNA twist angle and DNA diameter calculated from the simulation at 1 M NaCl. The unit of PMF is k_BT.

	32º-32.5º	32.5º-33º	33º-33.5º	33.5º-34º	34º-34.5º	34.5º-35º	35º-35.5º	35.5º-36º	36º-36.5º	36.5º-37º
1.99-2.00 nm	5.5276	4.0612	3.2589	3.5351	4.2844	5.9330				
1.98-1.99 nm	5.6707	3.7358	2.3248	2.0230	2.5153	3.6205	5.9330			
1.97-1.98 nm		3.8662	2.1580	1.2244	1.2262	1.9915	3.4735			
1.96-1.97 nm		4.4514	2.3221	1.0014	0.5011	0.6698	1.7283	3.8536		
1.95-1.96 nm		5.3453	2.8603	1.2715	0.2936	0.0578	0.5955	2.0351	4.6803	
1.94-1.95 nm			4.0309	2.0392	0.7003	0.0000	0.0386	1.0419	2.7426	
1.93-1.94 nm				3.2452	1.6536	0.5708	0.1446	0.6297	1.9760	4.0460
1.92-1.93 nm				4.8683	3.1667	1.6989	0.8800	0.8572	1.6817	3.3993
1.91-1.92 nm					4.9398	3.2520	2.1022	1.7373	2.1308	3.3453
1.9-1.91 nm						5.4630	3.9590	3.1922	2.9939	4.0767

Table S3. The twist-diameter coupling per bp at different salt concentrations from simulations.

NaCl (M)	k_{ω}^{bp} $(k_B T/\mathrm{deg}^2)$	k_D^{bp} $(k_B T/\mathrm{nm}^2)$	$k_{\omega D}^{bp}$ $(k_B T/\mathrm{deg}\cdot\mathrm{nm})$	ω_0 (degree)	D_0 (nm)
0.05	0.122±0.019	275±35	3.42±0.68	34.52	2.015
0.15	0.139±0.020	281±38	3.85±0.81	34.58	2.014
0.3	0.139±0.019	265±36	3.72±0.68	34.64	2.012
0.5	0.150±0.022	251±39	3.74±0.79	34.75	2.010
1	0.178±0.019	263±39	4.50±0.82	34.82	2.008

KCl (M)	k_{ω}^{bp} $(k_B T/\mathrm{deg}^2)$	k_D^{bp} $(k_B T/\mathrm{nm}^2)$	$k_{\omega D}^{bp}$ $(k_B T/\mathrm{deg}\cdot\mathrm{nm})$	ω_0 (degree)	D_0 (nm)
0.05	0.128±0.017	252±32	3.13±0.67	34.55	2.013
0.15	0.178±0.022	264±38	4.88±0.84	34.61	2.012
0.3	0.150±0.017	271±36	3.83±0.70	34.68	2.012
0.5	0.183±0.016	294±30	4.92±0.61	34.78	2.008
1	0.178±0.017	277±29	4.43±0.63	34.81	2.007

RbCl (M)	k_{ω}^{bp} $(k_B T/\mathrm{deg}^2)$	k_D^{bp} $(k_B T/\mathrm{nm}^2)$	$k_{\omega D}^{bp}$ $(k_B T/\mathrm{deg}\cdot\mathrm{nm})$	ω_0 (degree)	D_0 (nm)
0.05	0.167±0.018	269±34	4.46±0.79	34.70	2.010
0.15	0.183±0.019	291±35	5.00±0.71	34.74	2.009
0.3	0.161±0.015	295±29	5.31±0.60	34.83	2.007
0.5	0.167±0.014	312±24	4.67±0.48	34.88	2.004
1	0.172±0.015	318±31	4.52±0.62	34.93	2.003

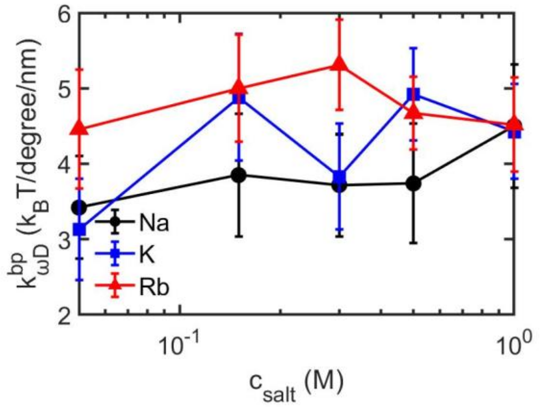

Fig. S6. **The twist-diameter coupling constant as a function of the concentration of NaCl, KCl or RbCl from simulations.**

S6. The roles of DNA contour length variation and twist-stretch coupling in salt effects

Lowering salt concentration enhances both inter-strand and longitudinal electrostatic repulsions, which should increase both DNA diameter and contour length and then affect DNA twist. The longitudinal electrostatic repulsion and the variation of DNA contour length play a minor role in salt-induced twist change, as indicated by several results presented below.

Our simulation results in **Figure S7** show that varying salt only slightly affects DNA contour length per base pair (referred as the rise in MD simulation). Compared to the rise, the diameter is much more significantly affected by salt variation.

The reason why the rise is weakly affected by salt is possibly due to the correlation between the rise, h and diameter, D. Such correlation is related to DNA volume, which is roughly determined as $V \approx D^2 h$. Changing DNA volume has an energy penalty, which may cause a negative correlation between the rise and diameter. Such correlation affects the variations of the rise and diameter upon salt change. Whether the rise or diameter is more varied by salt depends on the deformation pathway with the lowest energy cost. It turns out that the diameter variation dominates upon salt change, as indicated by **Figure S7**. It is quite likely that the increase of the diameter suppresses the increases of rise or reduces its extent due to the resistance of changing DNA volume. [Note that lowering c_{salt} increases DNA extension (or end-to-end distance) as shown by the experimental results in **Figure 1C** and **1D**. This result is contributed by the salt effects on both DNA contour length (*8*) and DNA persistence length (*8, 9*), while the effect on the persistence length appears to be much stronger than the effect on the contour length.]

Figure S8 shows the scatter plots for the twist, diameter, and rise (also referred to the stretch in line with literature). **Table S4** presents the correlations among the twist, diameter, and rise. As expected, we observed a weak negative correlation between the diameter and stretch. More importantly, twist-diameter correlation is much stronger than twist-stretch correlation. Note that the positive correlation between the twist and stretch at a fixed salt concentration shown in **Figure S8** and **Table S4** is consistent with twist-stretch coupling in previous studies (*1*).

It is interesting to compare the strengths of the twist-diameter coupling and the twist-stretch coupling using the following calculation. Driven by thermal motions, the magnitudes of the fluctuation, $\Delta\omega_t$ and ΔD_t, can be estimated through $\frac{1}{2} k_\omega^{\text{bp}} \Delta\omega_t^2 \approx k_B T$ and $\frac{1}{2} k_D^{\text{bp}} \Delta D_t^2 \approx k_B T$. The values of $\Delta\omega_t$ and ΔD_t lead to $P_{\text{couple}} = k_{\omega D}^{\text{bp}} \Delta\omega_t \Delta D_t \approx 2 k_{\omega D}^{\text{bp}} / \sqrt{k_\omega^{\text{bp}}\, k_D^{\text{bp}}}\, k_B T \approx 1.3\ k_B T$. It means that the fluctuations with magnitudes of $\Delta\omega_t$ and ΔD_t would result in twist-diameter coupling energy around 1.3 $k_B T$. A similar calculation for the twist-stretching coupling (*1, 21*) yields coupling energy of 0.2 $k_B T$, which is only about one-sixth of the one for the twist-diameter coupling.

In the main text, we express the DNA conformational free energy as $P(D, \omega)$. In principle, a rigorous expression should be $P(D, h, \omega)$, where h is the rise or stretch. However, we think the

current treatment of free energy in the form of $P(D, \omega)$ is more insightful due to several reasons. First, the twist is strongly correlated with the diameter and very weakly correlated with the rise. Second, only considering the contribution of ΔD to the twist can well reproduce salt-induced twist change. This is probably because the h variation upon salt change is negligible (cancellation of two effects mentioned above). Third, using the functional form of $P(D, h, \omega)$ blurs the physics of twist-diameter coupling and salt-induced twist change. Last but not least, the same twist-diameter coupling constant can well reproduce temperature-induced twist change.

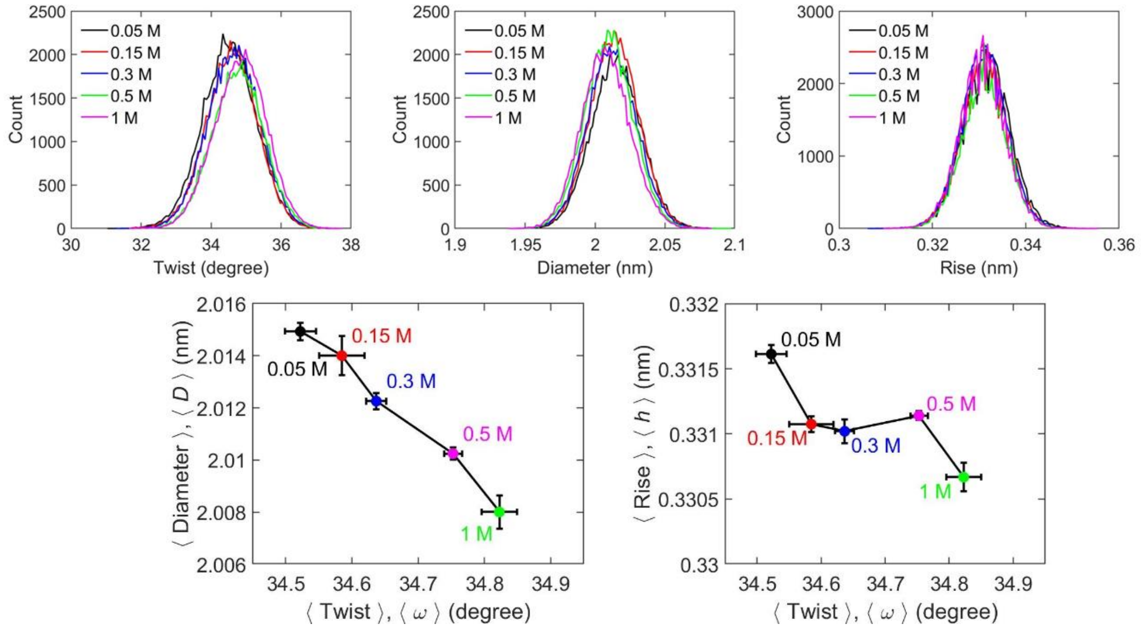

Fig. S7. **Effects of the NaCl concentration on the twist, diameter and rise (contour length per basepair).**

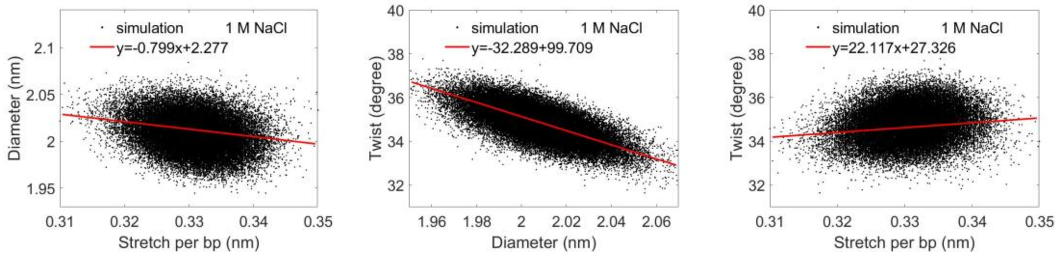

Fig. S8. **Scatter plots for twist, diameter and stretch from the simulation at 1 M NaCl.** Each dot corresponds to the data from one DNA conformation in the simulation.

Table S4. Correlation coefficients among twist, diameter and stretch from simulation results of various concentrations of NaCl, KCl and RbCl.

c_{salt} (NaCl)	Twist-diameter correlation coefficient	Twist-stretch correlation coefficient	Diameter-stretch correlation coefficient
0.05 M	-0.6996	0.1623	-0.2410
0.15 M	-0.6973	0.1661	-0.2430
0.3 M	-0.7045	0.1950	-0.2605
0.5 M	-0.6861	0.1725	-0.2548
1 M	-0.6922	0.1736	-0.2553

c_{salt} (KCl)	Twist-diameter correlation coefficient	Twist-stretch correlation coefficient	Diameter-stretch correlation coefficient
0.05 M	-0.6849	0.1750	-0.2643
0.15 M	-0.6806	0.1765	-0.2723
0.3M	-0.6951	0.1373	-0.2193
0.5 M	-0.6853	0.2167	-0.3058
1 M	-0.6712	0.1948	-0.2841

c_{salt} (RbCl)	Twist-diameter correlation coefficient	Twist-stretch correlation coefficient	Diameter-stretch correlation coefficient
0.05 M	-0.6687	0.1707	-0.2700
0.15 M	-0.6666	0.1788	-0.2759
0.3 M	-0.6645	0.1899	-0.2831
0.5 M	-0.6765	0.1804	-0.2713
1 M	-0.6658	0.1685	-0.2694

S7. Simulations with external forces to tune DNA diameter

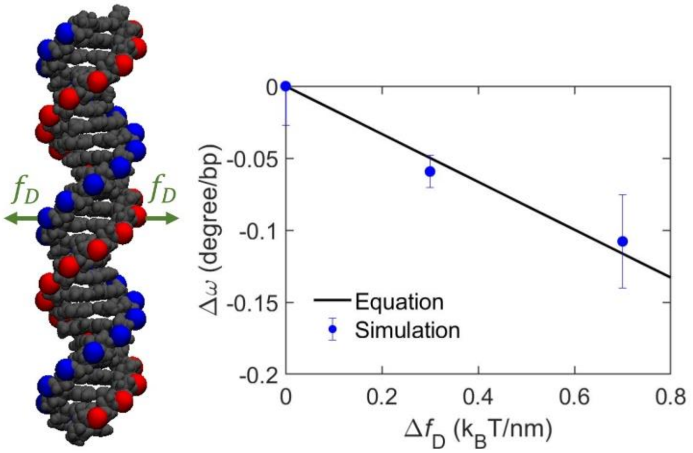

Fig. S9. **Effect of the external force on DNA twist.** DNA twist change as a function of the external force, Δf_D, that tends to increase DNA diameter. The simulation results (blue dots) agree with the prediction by Eq (5).

S8. Simulations with manual adjustment of the charge of the phosphate group

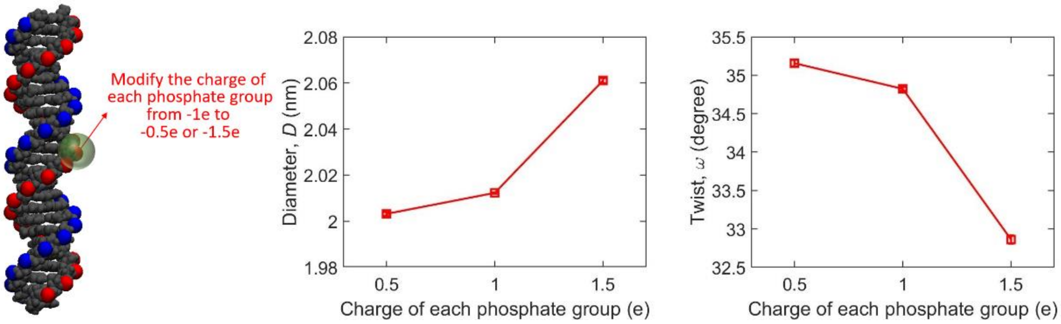

Fig. S10. **Effects of the artificial phosphate charge on DNA diameter and twist.** We artificially modified the charge of each phosphate group from -1e to -0.5e and -1.5e and measured the changes in DNA twist and diameter for the simulation at 1 M NaCl. The increase of the absolute value of the phosphate charge leads to the increase of DNA diameter and the decrease of DNA twist, while the decrease of the absolute value of the phosphate charge induces opposite effects.

S9. The effective twist rigidity for a DNA molecule with a relaxed diameter

We can calculate the effective twist rigidity for a DNA molecule with a relaxed diameter from k_ω^{bp}, k_D^{bp} and $k_{\omega D}^{\text{bp}}$. Eq (2) can be reformed as

$$P = \frac{1}{2} k_\omega^{\text{bp}} (\Delta\omega)^2 + \frac{1}{2} k_D^{\text{bp}} (\Delta D)^2 + k_{\omega D}^{\text{bp}} \Delta\omega \Delta D$$

$$= \frac{1}{2} k_D^{\text{bp}} \left(\Delta D - \frac{k_{\omega D}^{\text{bp}}}{k_D^{\text{bp}}} \Delta\omega \right)^2 - \frac{1}{2} \frac{\left(k_{\omega D}^{\text{bp}}\right)^2}{k_D^{\text{bp}}} (\Delta\omega)^2 + \frac{1}{2} k_\omega^{\text{bp}} (\Delta\omega)^2$$

$$= \frac{1}{2} k_D^{\text{bp}} \left(\Delta D - \frac{k_{\omega D}^{\text{bp}}}{k_D^{\text{bp}}} \Delta\omega \right)^2 + \frac{1}{2} \left[k_\omega^{\text{bp}} - \frac{\left(k_{\omega D}^{\text{bp}}\right)^2}{k_D^{\text{bp}}} \right]^2 (\Delta\omega)^2 \quad \text{(S16)}$$

For every twist angle Δω, the DNA diameter will be relaxed toward $\Delta D = \frac{k_{\omega D}^{\text{bp}}}{k_D^{\text{bp}}} \Delta\omega$ to minimize the first term, and then the energy cost is the second term $\frac{1}{2} \left[k_\omega^{\text{bp}} - \frac{\left(k_{\omega D}^{\text{bp}}\right)^2}{k_D^{\text{bp}}} \right]^2 (\Delta\omega)^2$. So, the effective twist rigidity is

$$\tilde{k}_\omega^{\text{bp}} = k_\omega^{\text{bp}} - \frac{\left(k_{\omega D}^{\text{bp}}\right)^2}{k_D^{\text{bp}}} \approx 0.103\ k_B T/deg^2\ . \quad \text{(S17)}$$

While we define the twist rigidity for a base pair, another definition is $P = \frac{1}{2} \frac{C}{L} (\Delta\omega)^2$, where P is the energy cost, C is the twist rigidity, and L is the DNA length. Then, $C = \tilde{k}_\omega^{\text{bp}} \times L_{bp}$, where $L_{bp} \approx 0.338$ nm. After using the unit conversion 1 rad = 180/π deg and 1 k_BT = 4.114 pN nm, eventually, we have $C = 0.103 \times \left(\frac{180}{\pi}\right)^2 \times 4.114 \times 0.338$ = 470 pN nm^2.

S10. The effect of Δf_D on k_ω^{bp}, k_D^{bp} and $k_{\omega D}^{\mathrm{bp}}$

Eq (4) can be reformed as

$$P(c_{\mathrm{salt}}) \approx -\Delta f_D \Delta D + \frac{1}{2} k_\omega^{\mathrm{bp}} (\Delta\omega)^2 + \frac{1}{2} k_D^{\mathrm{bp}} (\Delta D)^2 + k_{\omega D}^{\mathrm{bp}} \Delta\omega \Delta D$$

$$= \frac{1}{2} k_\omega^{\mathrm{bp}} \left(\Delta\omega'\right)^2 + \frac{1}{2} k_D^{\mathrm{bp}} \left(\Delta D'\right)^2 + k_{\omega D}^{\mathrm{bp}} \Delta\omega' \Delta D' - \frac{1}{2} \frac{k_\omega^{\mathrm{bp}}}{k_\omega^{\mathrm{bp}} k_D^{\mathrm{bp}} - \left(k_{\omega D}^{\mathrm{bp}}\right)^2} (\Delta f_D)^2$$

with $$\Delta\omega' = \Delta\omega + \frac{k_{\omega D}^{\mathrm{bp}}}{k_\omega^{\mathrm{bp}} k_D^{\mathrm{bp}} - \left(k_{\omega D}^{\mathrm{bp}}\right)^2} \Delta f_D$$

$$\Delta D' = \Delta D - \frac{k_\omega^{\mathrm{bp}}}{k_\omega^{\mathrm{bp}} k_D^{\mathrm{bp}} - \left(k_{\omega D}^{\mathrm{bp}}\right)^2} \Delta f_D \qquad \text{(S18)}$$

The above equation indicates that the addition of $-\Delta f_D \Delta D$ does not affect k_ω^{bp}, k_D^{bp} and $k_{\omega D}^{\mathrm{bp}}$, but just shifts the position and value of the free-energy minimum.

S11. The prevalence of twist-diameter coupling in salt-induced twist change, temperature-induced twist change, and stretch-induced twist change

We performed additional MT experiments and MD simulations to analyze the twist-diameter coupling in temperature-induced twist change, as shown in **Figure S11**. Our experimental and simulation results of temperature-induced twist change agree with each other and quantitatively reproduced the previous experimental and simulation results (*6*), as shown in **Figure 5B**.

Our new analysis revealed the mechanism for temperature-induced twist change: increasing temperature causes the swelling of DNA diameter, which leads to the decrease of DNA twist through the twist-diameter coupling. We find that equilibrium DNA diameter is resulted from the competition of inter-strand attractions and entropy, which tend to shrink and swell DNA diameter, respectively. The increase in temperature enhances the contribution of entropy and hence swells DNA diameter. The mechanism is confirmed by the following quantitative results. Our MD simulations yield PMF with respect to the diameter under various temperatures (**Figure S11(a)**), which allows us to separate the contributions of interaction energies U and conformational entropy S to DNA free energy:

$$F(D) = U(D) - TS(D) \qquad \text{(S19)}$$

where $U(D)$ is the internal energy and $S(D)$ is the entropy, both as a function of diameter. Taking the PMF of 22 °C as a reference, we subtract it from each PMF at other temperatures (7, 17, 37, and 47 °C) to give the PMF difference (**Figure S11(b)**).

$$F(7°\mathrm{C}) - F(22°\mathrm{C}) = 15S(D) + a$$

$$F(17°\mathrm{C}) - F(22°\mathrm{C}) = 5S(D) + b$$

$$F(37°\mathrm{C}) - F(22°\mathrm{C}) = -15S(D) + c$$

$$F(47°\mathrm{C}) - F(22°\mathrm{C}) = -25S(D) + d \qquad (S20)$$

where a, b, c and d are constants that are independent of D. These constants are due to the fact that there is an arbitrary constant when calculating PMF from the density of states in simulations. Eq (S20) eliminates $U(D)$ by calculating the differences between PMFs. Only $S(D)$ remains. Dividing each equation in Eq (S20) by the prefactor of $S(D)$, we obtain multiple sets of $S(D)$ plus a constant:

$$[F(7°\mathrm{C}) - F(22°\mathrm{C})]/15 = S(D) + a/15$$

$$[F(17°\mathrm{C}) - F(22°\mathrm{C})]/5 = S(D) + b/5$$

$$[F(37°\mathrm{C}) - F(22°\mathrm{C})]/(-15) = S(D) + c/(-15)$$

$$[F(47°\mathrm{C}) - F(22°\mathrm{C})]/(-25) = S(D) + d/(-25) \qquad (S21)$$

Note that the constants do not matter, because we just need the change of S with D instead of the absolute value of S. The multiple sets of $S(D)$ are averaged to reduce the statistical error. As shown in **Figure S11(c)**, we find that extracted DNA conformational entropy, $S(D)$, indeed increases with the diameter linearly, specifically in the range of $D \in [2.00,2.03]$ nm. To confirm this trend, we calculated the root-mean-square deviation (RMSD) of DNA conformations as a function of the diameter from MD simulations (**Figure S11(d)**). As expected, RMSD increases with the diameter. This is reasonable considering that a larger diameter corresponds to more space for DNA fluctuation. To connect the diameter-dependent entropy $S(D)$ with temperature-dependent twist change $\omega(T)$, we performed the following calculation. The change in temperature, ΔT, exerts an effective force on DNA diameter, $\Delta f_T(\Delta T)$:

$$\Delta f_T = -\frac{\partial \Delta F}{\partial D} = \frac{\partial (S\Delta T)}{\partial D} = \Delta T \frac{\partial S}{\partial D} \approx k_{SD} \times \Delta T \qquad \text{with} \qquad k_{SD} \equiv \frac{\partial S}{\partial D} \approx 0.146 \ \text{kJ/(mol} \cdot \text{K} \cdot \text{nm)} \tag{S22}$$

The value of $\partial S/\partial D \approx 0.146$ kJ/(mol·K·nm) was obtained from the simulation result in **Figure 5D**. In the main manuscript, we have obtained the relationship between the effective force that tends to change the diameter and twist. Accordingly, we have:

$$\Delta\omega_{\text{bp}}(\Delta T) = \frac{-k_{\omega D}^{\text{bp}}}{k_{\omega}^{\text{bp}} k_{D}^{\text{bp}} - \left(k_{\omega D}^{\text{bp}}\right)^2} \Delta f_T = \frac{-k_{\omega D}^{\text{bp}}}{k_{\omega}^{\text{bp}} k_{D}^{\text{bp}} - \left(k_{\omega D}^{\text{bp}}\right)^2} k_{SD} \Delta T \tag{S23}$$

It means the temperature-dependent twist change has a coefficient k_T:

$$k_T^{\text{bp}} \equiv \frac{\Delta\omega_{\text{bp}}}{\Delta T} = \frac{-k_{\omega D}^{\text{bp}}}{k_{\omega}^{\text{bp}} k_{D}^{\text{bp}} - \left(k_{\omega D}^{\text{bp}}\right)^2} k_{SD} \tag{S24}$$

At T=22 ^{o}C , using the relationship 1 $k_B T \approx 2.454$ kJ/mol, we made the conversion: $k_{SD} \approx$ 0.146 kJ/(mol·^{o}C·nm) $\approx$ 0.0595 k_BT/(°C · nm). With $\frac{-k_{\omega D}^{\text{bp}}}{k_{\omega}^{\text{bp}} k_{D}^{\text{bp}} - \left(k_{\omega D}^{\text{bp}}\right)^2} \approx -0.166 \text{ deg} \cdot \text{nm}/k_B$T.

Eventually, we predict

$$k_T^{\text{bp}} \approx -0.166 \times 0.0595 \text{ deg/°C} \approx -0.01 \text{ deg/°C}. \tag{S25}$$

The above coefficient agrees with our experimental results, as shown in **Figure 5B**.

In addition to salt- and temperature-induced twist changes, stretch-induced twist change is likely to be related to twist-diameter coupling. As illustrated in **Figure S12**, one mechanism proposed by Gore et al. (*1*) for stretch-induced twist change consists of two steps: (i) stretching DNA causes the shrinking of DNA diameter due to DNA volume conservation; (ii) the shrinking of DNA diameter is transduced to DNA overwinding. The second step implies the twist-diameter coupling, i.e., the transduction from diameter change to twist change. This proposed mechanism is essentially based on volume conservation (relating stretch and diameter) and twist-diameter

coupling (relating diameter and twist). Such mechanism is supported by our simulation results in **Figure S13**.

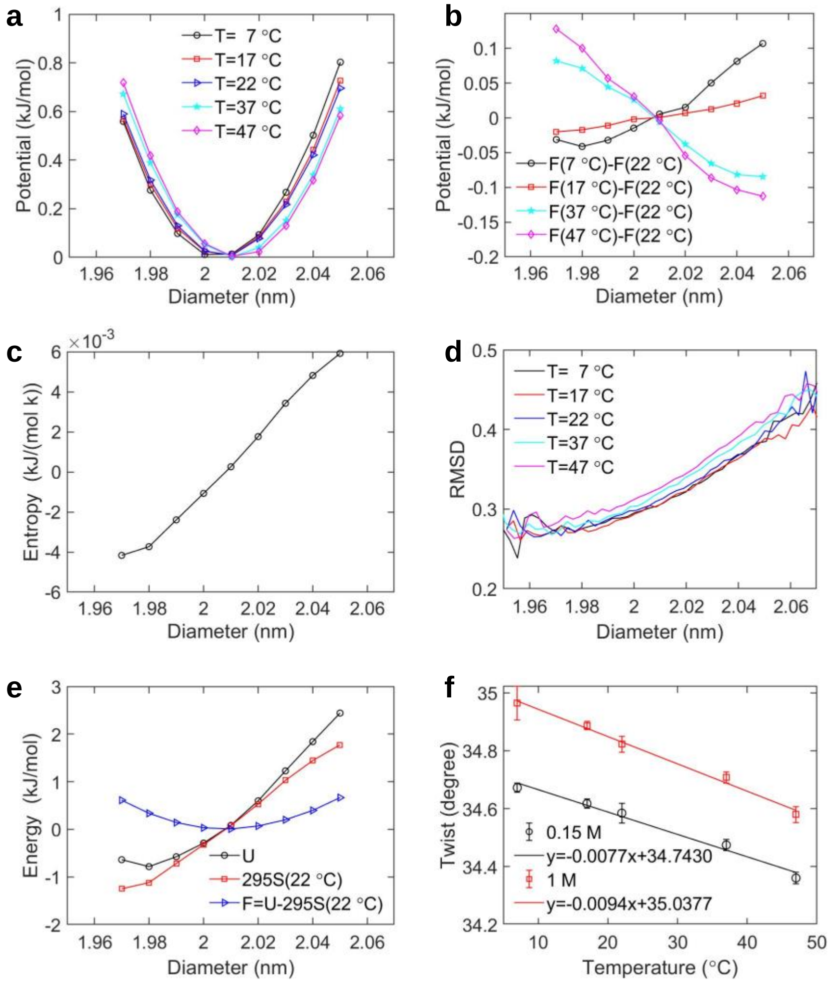

Fig. S11. **Analysis of simulation results of temperature-induced twist changes.** (**a**) Simulation results of DNA diameter as a function of temperature. (**b**) Free energy difference as a function of diameter for independent temperatures at 1 M NaCl. (**c**) Extracted entropy as a function of the diameter for independent temperatures at 1 M NaCl. (**d**) DNA conformational fluctuations as a function of the diameter from 1 M NaCl. (**e**) Simulation results of interaction energy $U(D)$, rescaled entropy $295K \times S(D)$, and free energy as a function of the diameter at 1 M NaCl. (**f**) Twist as a function of temperature from simulation and their fitted lines.

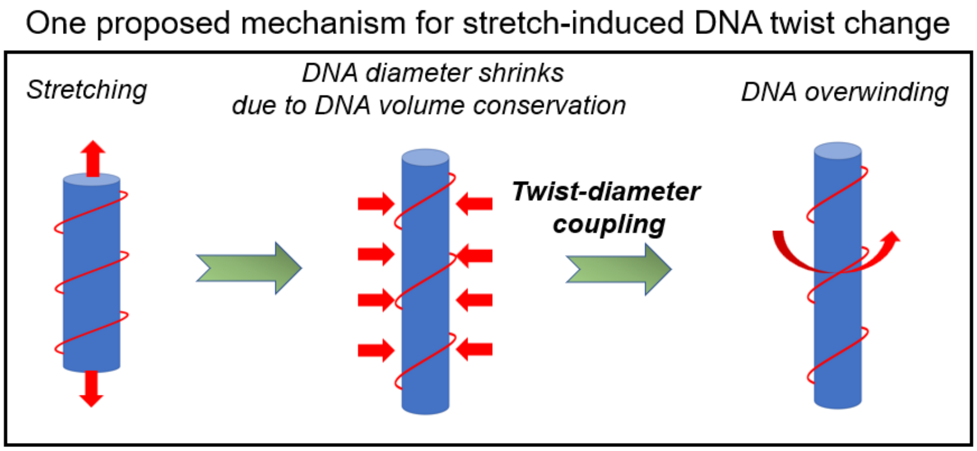

Fig. S12. One proposed mechanism for stretch-induced DNA twist change by Gore et al [Nature (2006) 442, 836]. This proposal mechanism implicitly includes twist-diameter coupling.

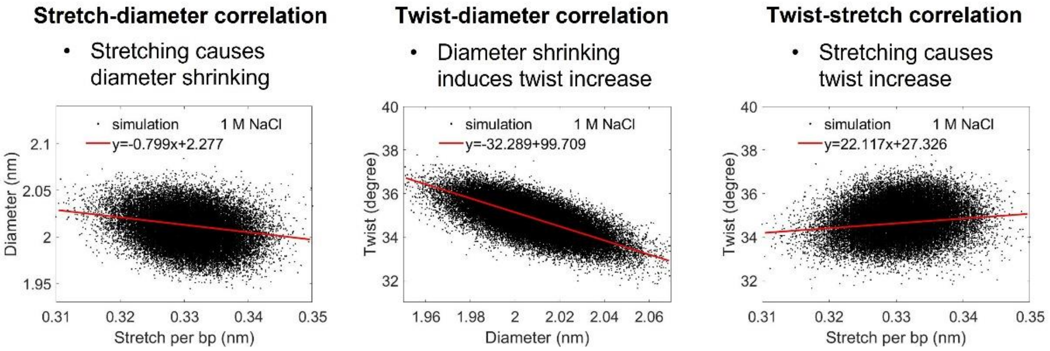

Fig. S13. Simulation results of stretch-diameter correlation, twist-diameter correlation, and twist-stretch correlation. Combined with stretch-diameter correlation, twist-diameter coupling can lead to twist-stretch coupling.

S12. Calculation of the twist-diameter coupling constant from the stretch modulus of DNA backbone length

In this section, we estimate the twist-diameter coupling constant from the stretch modulus of DNA backbone length, as shown in **Figure S14**. Gore al et al. obtained the stretch modulus of DNA backbone strand as $S_h \approx 965$ pN. Due to this stretch modulus, the variations of twist and diameter change the backbone contour length s and require an energetic cost, which leads to the twist-diameter coupling. Based on this idea, we can convert the stretch modulus to the twist-diameter coupling constant. We denote the contour length of DNA backbone strand per bp as s and the rise per bp as h. Then, one has

$$s \approx \sqrt{(D\omega/2)^2 + h^2}\ . \qquad \text{(S26)}$$

Small perturbations in diameter ΔD and in twist $\Delta\omega$ leads to the change in s:

$$\Delta s \approx \frac{D_0\omega_0^2}{4s_0}\Delta D + \frac{D_0^2\omega_0}{4s_0}\Delta\omega\ . \qquad \text{(S27)}$$

The change Δs corresponds to the energetic penalty:

$$\Delta E_{backbone}^{bp} \approx 2 \times \frac{1}{2}\frac{S_h}{s_0}(\Delta s)^2. \qquad \text{(S28)}$$

The multiplication of 2 in the above equations is due to the fact that every base pair contains two backbone strands (DNA is a double helix).

To obtain the twist-diameter coupling constant, we only need to know the crossing term related to $\Delta D\Delta\omega$. Then, substituting Eq (S25) into Eq (S26) yields

$$\Delta E_{backbone}^{bp} \approx \cdots + \left(\frac{S_h}{s_0}\right)\frac{D_0^3\omega_0^3}{8s_0^2}\Delta D\Delta\omega + \cdots. \qquad \text{(S29)}$$

We obtain the relationship between the twist-diameter coupling and the stretch modulus:

$$k_{\omega D}^{bp} = \frac{S_h D_0^3\omega_0^3}{8s_0^3}\ . \qquad \text{(S30)}$$

Substituting the parameters $S_h = 965$ pN, $D_0 = 2.008$ nm, $\omega_0 = 34.82^o = 0.6077$ rad, $h_0 = 0.331$ nm, $s_0 = 0.694$ nm into Eq (S28), we have

$$k_{\omega D}^{bp} = \frac{S_h D_0^3\omega_0^3}{8s_0^3} \approx 656\, pN/rad \approx 159\ k_BT/(nm \cdot rad) \approx 2.8\ k_BT/(deg \cdot nm).$$

The calculated coupling constant 2.8 $k_BT/(deg \cdot nm)$ agrees fairly well with 4.5 $k_BT/(deg \cdot nm)$ obtained from MD simulation. The small difference between the two values is not surprising considering that they are derived from two types of DNA deformations: stretching and varying salt concentration. In particular, the above calculation ignores the variation of h. In the case of lowering c_{salt}, both D and h should increase. The increase of h should cause an

additional decrease in ω based on Eq (S24), which makes the twist-diameter coupling appear stronger than expected. This factor may be the reason why the estimated $k_{\omega D}^{bp}$ above is smaller than the one calculated from MD simulation.

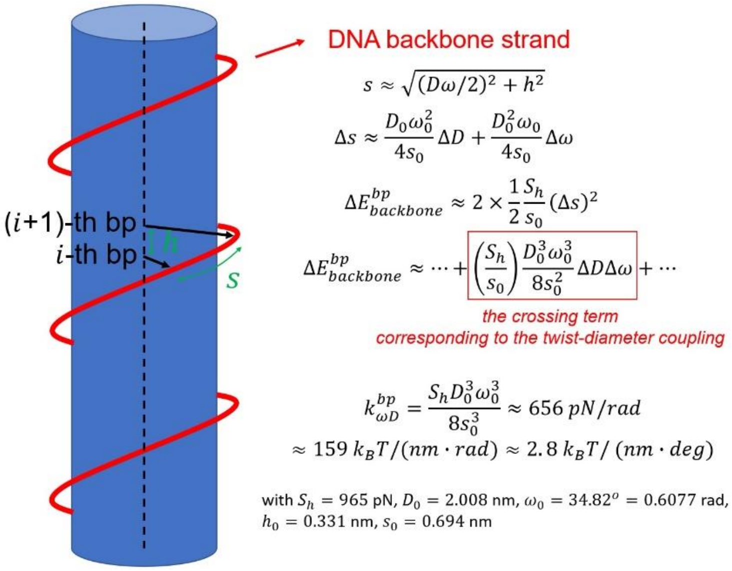

Fig. S14. **Derivation of the twist-diameter coupling from the restraint of DNA backbone stand.**

S13. Evidence of diameter variation and twist-diameter coupling in salt effects and temperature effects

Here, we summarize the evidence that salt- and temperature-induced twist changes are mainly mediated by diameter variation and twist-diameter coupling. At first, it is worth pointing out that DNA diameter quantifies phosphate-phosphate distance that tunes electrostatic interactions in salt effects and also quantifies inter-strand distance that tunes DNA conformational entropy in temperature effect, as illustrated in **Figure S15**.

Figure S16 summarizes abundant evidence to demonstrate that salt-induced DNA twist change is mainly through the pathway: changing salt → diameter variation → twist variation.

Figure S17 addresses three questions that are involved in our interpretation of the pathways in salt-induced DNA twist change.

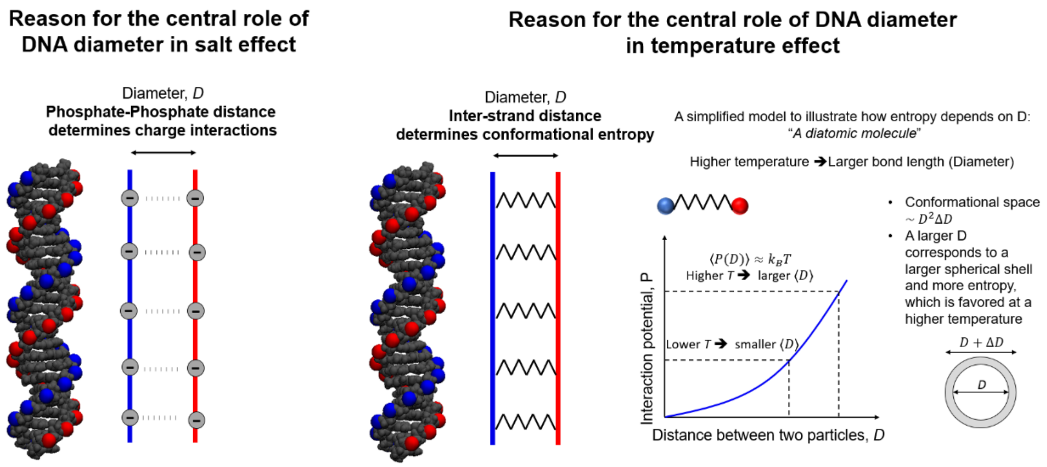

Fig. S15. **Central roles of diameter in salt effect and temperature effect.**

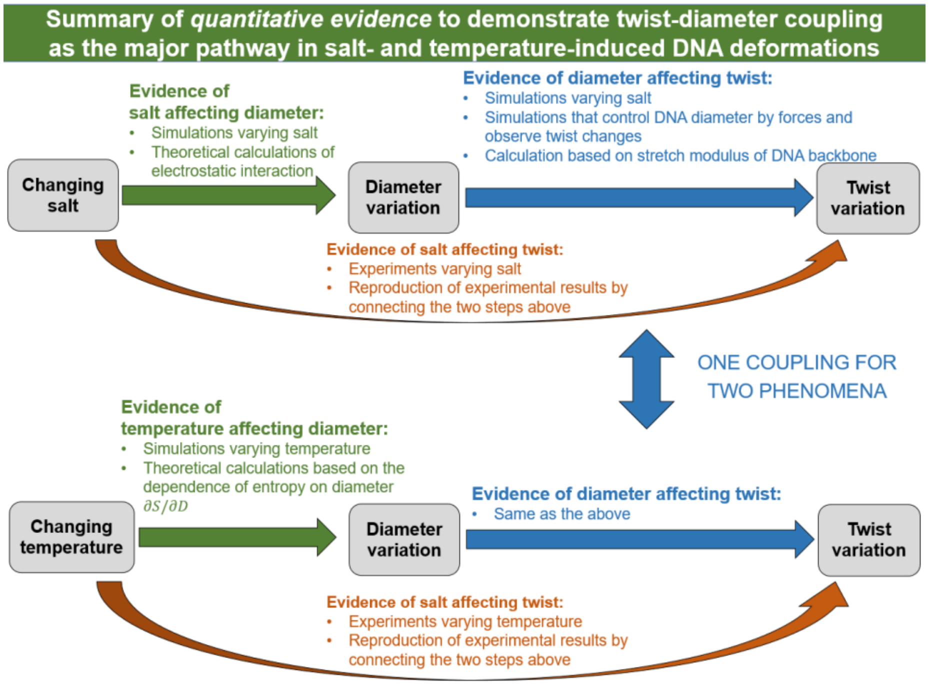

Fig. S16. Summary of evidence to demonstrate twist-diameter coupling and the central roles of diameter variation in salt- and temperature-induced twist changes.

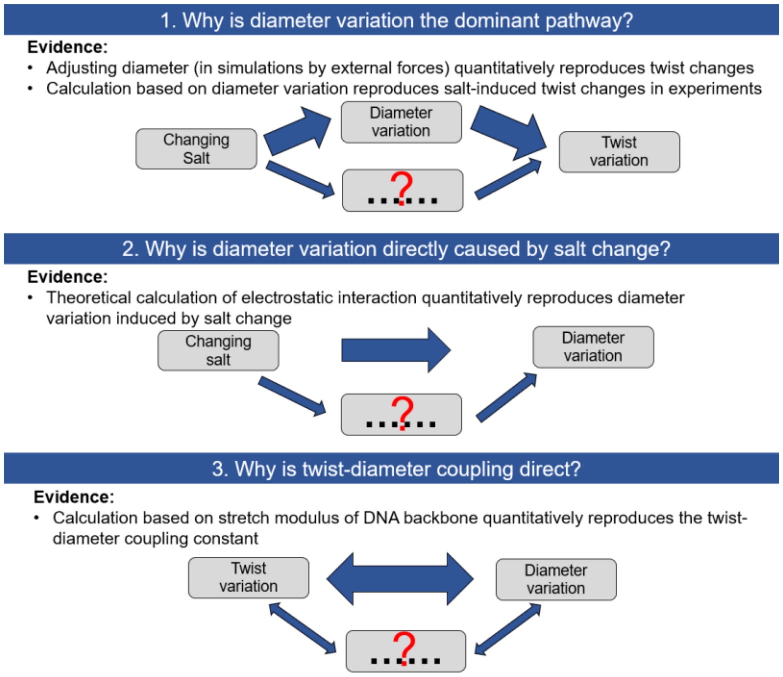

Fig. S17. Evidence that supports our interpretation of the pathways in DNA deformations.

S14. Conversion from k_D^{bp} to DNA Young's modulus

Figure S18 presents the calculation procedure from k_D^{bp} to DNA Young's modulus. Our simulation value of $k_D^{\mathrm{bp}} = 263\ k_B T/nm^2$ corresponds to DNA Young's modulus of 3.2×10^8 Pa, which agrees with the experimental value of 3.46×10^8 Pa by Smith, Cui, and Bustamante (*38*).

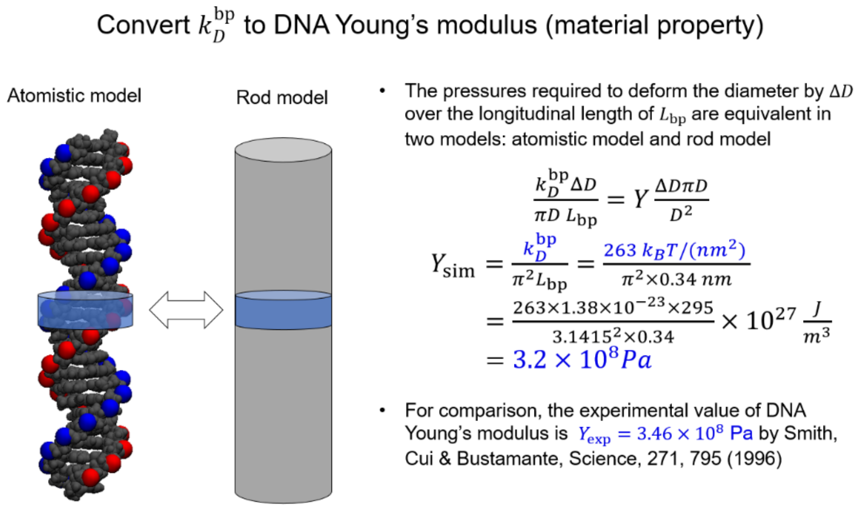

Fig. S18. Conversion from k_D^{bp} to DNA Young's modulus.

S15. Sequence dependence of salt- and temperature-induced DNA twist changes in experiments and simulations

Figure S19 presents our experimental results of the salt-induced and temperature-induced DNA twist changes for three constructs: (i) 13k bp GC content 43%; (ii) 20 kbp GC content 55%; (iii) 6 kbp GC content 57%. The results for these three DNA molecules agree well with each other, which suggests that the salt-induced and temperature-induced DNA twist changes are not caused by the specific DNA construct used in our experiments.

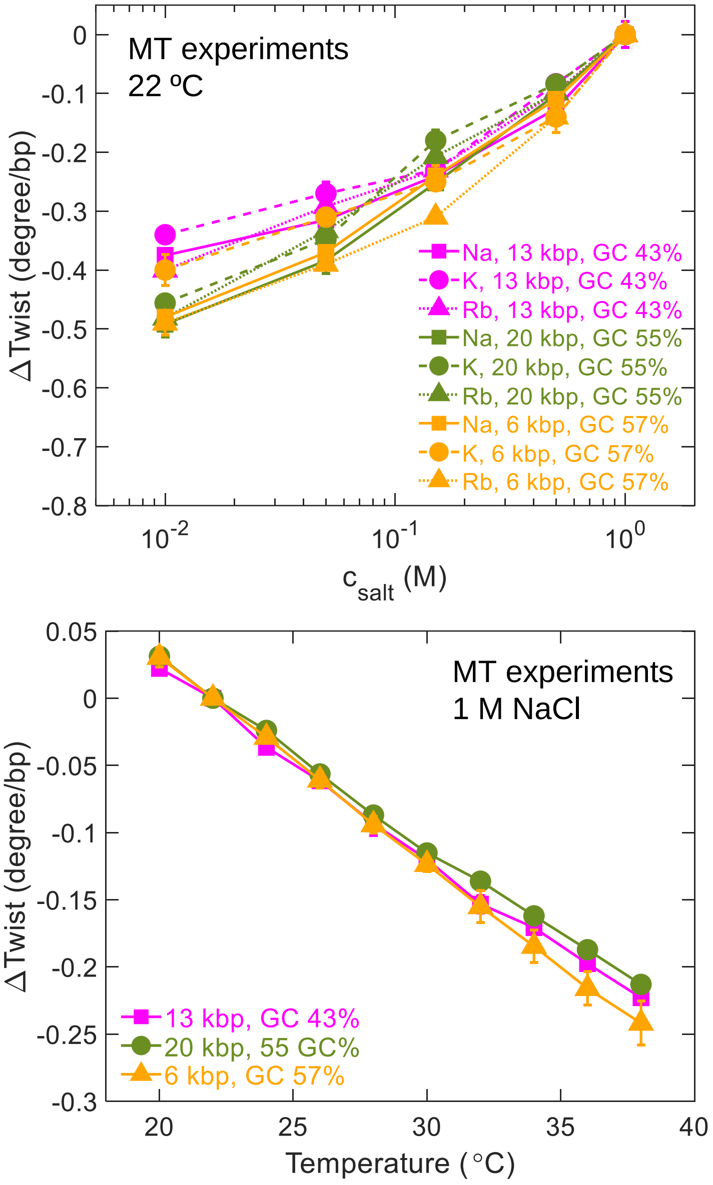

Fig. S19. Comparison of experimental results for different DNA constructs. Our MT experimental results of salt-induced and temperature-induced DNA twist changes for three different DNA constructs: (i) 13 kbp, 43% GC; (ii) 20 kbp, 55% GC; (iii) 6 kbp, 57%. For each DNA construct at each salt condition or temperature, the experimental data obtained from more than four molecules were plotted as data points and error bars.

REFERENCES

1. J. Gore, Z. Bryant, M. Nöllmann, M. U. Le, N. R. Cozzarelli, C. Bustamante, DNA overwinds when stretched. *Nature* **442**, 836–839 (2006).

2. P. Gross, N. Laurens, L. B. Oddershede, U. Bockelmann, E. J. G. Peterman, G. J. L. Wuite, Quantifying how DNA stretches, melts and changes twist under tension. *Nat. Phys.* **7**, 731–736 (2011).

3. J. F. Marko, E. D. Siggia, Stretching DNA. *Macromolecules* **28**, 8759–8770 (1995).

4. G. A. King, F. Burla, E. J. Peterman, G. J. L. Wuite, Supercoiling DNA optically. *Proc. Natl. Acad. Sci. U.S.A.* **116**, 26534–26539 (2019).

5. G. A. King, E. J. G. Peterman, G. J. L. Wuite, Unravelling the structural plasticity of stretched DNA under torsional constraint. *Nat. Commun.* **7**, 11810 (2016).

6. F. Kriegel, C. Matek, T. Dršata, K. Kulenkampff, S. Tschirpke, M. Zacharias, F. Lankaš, J. Lipfert, The temperature dependence of the helical twist of DNA. *Nucleic Acids Res.* **46**, 7998–8009 (2018).

7. H. Dohnalová, T. Dršata, J. Šponer, M. Zacharias, J. Lipfert, F. Lankaš, Compensatory mechanisms in temperature dependence of DNA double helical structure: Bending and elongation. *J. Chem. Theory Comput.* **16**, 2857–2863 (2020).

8. H. Fu, C. Zhang, X.-W. Qiang, Y.-J. Yang, L. Dai, Z.-J. Tan, X.-H. Zhang, Opposite effects of high-valent cations on the elasticities of DNA and RNA duplexes revealed by magnetic tweezers. *Phys. Rev. Lett.* **124**, 058101 (2020).

9. S. Guilbaud, L. Salomé, N. Destainville, M. Manghi, C. Tardin, Dependence of DNA persistence length on ionic strength and ion type. *Phys. Rev. Lett.* **122**, 028102 (2019).

10. O. D. Broekmans, G. A. King, G. J. Stephens, G. J. L. Wuite, DNA twist stability changes with magnesium(2+) concentration. *Phys. Rev. Lett.* **116**, 258102 (2016).

11. F. Kriegel, N. Ermann, R. Forbes, D. Dulin, N. H. Dekker, J. Lipfert, Probing the salt dependence of the torsional stiffness of DNA by multiplexed magnetic torque tweezers. *Nucleic Acids Res.* **45**, 5920–5929 (2017).

12. J. R. Wenner, M. C. Williams, I. Rouzina, V. A. Bloomfield, Salt dependence of the elasticity and overstretching transition of single DNA molecules. *Biophys. J.* **82**, 3160–3169 (2002).

13. S. Kim, E. Broströmer, D. Xing, J. Jin, S. Chong, H. Ge, S. Wang, C. Gu, L. Yang, Y. Q. Gao, X.-d. Su, Y. Sun, X. S. Xie, Probing allostery through DNA. *Science* **339**, 816–819 (2013).

14. L. Shokri, B. Marintcheva, M. Eldib, A. Hanke, I. Rouzina, M. C. Williams, Kinetics and thermodynamics of salt-dependent T7 gene 2.5 protein binding to single- and double-stranded DNA. *Nucleic Acids Res.* **36**, 5668–5677 (2008).

15. C. J. Lim, L. J. Kenney, J. Yan, Single-molecule studies on the mechanical interplay between DNA supercoiling and H-NS DNA architectural properties. *Nucleic Acids Res.* **42**, 8369–8378 (2014).

16. A. M. Maier, W. Bae, D. Schiffels, J. F. Emmerig, M. Schiff, T. Liedl, Self-assembled DNA tubes forming helices of controlled diameter and chirality. *ACS Nano* **11**, 1301–1306 (2017).

17. H. Dietz, S. M. Douglas, W. M. Shih, Folding DNA into twisted and curved nanoscale shapes. *Science* **325**, 725–730 (2009).

18. T. Lionnet, S. Joubaud, R. Lavery, D. Bensimon, V. Croquette, Wringing out DNA. *Phys. Rev. Lett.* **96**, 178102 (2006).

19. J. Lipfert, G. M. Skinner, J. M. Keegstra, T. Hensgens, T. Jager, D. Dulin, M. Köber, Z. Yu, S. P. Donkers, F.-C. Chou, R. Das, N. H. Dekker, Double-stranded RNA under force and torque: Similarities to and striking differences from double-stranded DNA. *Proc. Natl. Acad. Sci. U.S.A.* **111**, 15408–15413 (2014).

20. K. Liebl, T. Drsata, F. Lankas, J. Lipfert, M. Zacharias, Explaining the striking difference in twist-stretch coupling between DNA and RNA: A comparative molecular dynamics analysis. *Nucleic Acids Res.* **43**, 10143–10156 (2015).

21. A. Marin-Gonzalez, J. G. Vilhena, R. Perez, F. Moreno-Herrero, Understanding the mechanical response of double-stranded DNA and RNA under constant stretching forces using all-atom molecular dynamics. *Proc. Natl. Acad. Sci. U.S.A.* **114**, 7049–7054 (2017).

22. G. S. Manning, Theoretical evidence for the coupling of winding to compression in the solution conformation of duplex DNA. *Biopolymers* **20**, 2337–2350 (1981).

23. E. Skoruppa, S. K. Nomidis, J. F. Marko, E. Carlon, Bend-induced twist waves and the structure of nucleosomal DNA. *Phys. Rev. Lett.* **121**, 088101 (2018).

24. S. K. Nomidis, F. Kriegel, W. Vanderlinden, J. Lipfert, E. Carlon, Twist-bend coupling and the torsional response of double-stranded DNA. *Phys. Rev. Lett.* **118**, 217801 (2017).

25. C. Gu, J. Zhang, Y. I. Yang, X. Chen, H. Ge, Y. Sun, X. Su, L. Yang, S. Xie, Y. Q. Gao, DNA structural correlation in short and long ranges. *J. Phys. Chem. B* **119**, 13980–13990 (2015).

26. P. Anderson, W. Bauer, Supercoiling in closed circular DNA: Dependence upon ion type and concentration. *Biochemistry* **17**, 594–601 (1978).

27. K. Andresen, R. Das, H. Y. Park, H. Smith, L. W. Kwok, J. S. Lamb, E. Kirkland, D. Herschlag, K. Finkelstein, L. Pollack, Spatial distribution of competing ions around DNA in solution. *Phys. Rev. Lett.* **93**, 248103 (2004).

28. A. Savelyev, A. D. MacKerell Jr., Competition among Li^+, Na^+, K^+, and Rb^+ monovalent ions for DNA in molecular dynamics simulations using the additive CHARMM36 and drude polarizable force fields. *J. Phys. Chem. B* **119**, 4428–4440 (2015).

29. R. Lavery, J. H. Maddocks, M. Pasi, K. Zakrzewska, Analyzing ion distributions around DNA. *Nucleic Acids Res.* **42**, 8138–8149 (2014).

30. X. Qiu, D. C. Rau, V. A. Parsegian, L. T. Fang, C. M. Knobler, W. M. Gelbart, Salt-dependent DNA-DNA spacings in intact bacteriophage λ reflect relative importance of DNA self-repulsion and bending energies. *Phys. Rev. Lett.* **106**, 028102 (2011).

31. M. L. Sushko, D. G. Thomas, S. A. Pabit, L. Pollack, A. V. Onufriev, N. A. Baker, The role of correlation and solvation in ion interactions with B-DNA. *Biophys. J.* **110**, 315–326 (2016).

32. L.-Z. Sun, Y. Zhou, S.-J. Chen, Predicting monovalent ion correlation effects in nucleic acids. *ACS Omega* **4**, 13435–13446 (2019).

33. R. S. Mathew-Fenn, R. Das, P. A. B. Harbury, Remeasuring the double helix. *Science* **322**, 446–449 (2008).

34. D. Van Der Spoel, E. Lindahl, B. Hess, G. Groenhof, A. E. Mark, H. J. C. Berendsen, GROMACS: Fast, flexible, and free. *J. Comput. Chem.* **26**, 1701–1718 (2005).

35. M. Zgarbová, J. Šponer, M. Otyepka, T. E. Cheatham III, R. Galindo-Murillo, P. Jurečka, Refinement of the sugar–phosphate backbone torsion beta for AMBER force fields improves the description of Z- and B-DNA. *J. Chem. Theory Comput.* **11**, 5723–5736 (2015).

36. R. Lavery, M. Moakher, J. H. Maddocks, D. Petkeviciute, K. Zakrzewska, Conformational analysis of nucleic acids revisited: Curves+. *Nucleic Acids Res.* **37**, 5917–5929 (2009).

37. J. F. Marko, DNA under high tension: Overstretching, undertwisting, and relaxation dynamics. *Phys. Rev. E* **57**, 2134–2149 (1998).

38. S. B. Smith, Y. Cui, C. Bustamante, Overstretching B-DNA: The elastic response of individual double-stranded and single-stranded DNA molecules. *Science* **271**, 795–799 (1996).

39. Z. Bryant, M. D. Stone, J. Gore, S. B. Smith, N. R. Cozzarelli, C. Bustamante, Structural transitions and elasticity from torque measurements on DNA. *Nature* **424**, 338–341 (2003).

40. M. Y. Sheinin, M. D. Wang, Twist-stretch coupling and phase transition during DNA supercoiling. *Phys. Chem. Chem. Phys.* **11**, 4800–4803 (2009).

41. J. D. Moroz, P. Nelson, Entropic elasticity of twist-storing polymers. *Macromolecules* **31**, 6333–6347 (1998).

42. G. S. Manning, Counterion condensation on a helical charge lattice. *Macromolecules* **34**, 4650–4655 (2001).

43. G. S. Manning, Electrostatic free energy of the DNA double helix in counterion condensation theory. *Biophys. Chem.* **101-102**, 461–473 (2002).

44. G. S. Manning, The response of DNA length and twist to changes in ionic strength. *Biopolymers* **103**, 223–226 (2015).

45. I. Ivani, P. D. Dans, A. Noy, A. Pérez, I. Faustino, A. Hospital, J. Walther, P. Andrio, R. Goñi, A. Balaceanu, G. Portella, F. Battistini, J. L. Gelpí, C. González, M. Vendruscolo, C. A. Laughton, S. A. Harris, D. A. Case, M. Orozco, Parmbsc1: A refined force field for DNA simulations. *Nat. Methods* **13**, 55–58 (2016).

46. Z. Yu, D. Dulin, J. Cnossen, M. Köber, M. M. van Oene, O. Ordu, B. A. Berghuis, T. Hensgens, J. Lipfert, N. H. Dekker, A force calibration standard for magnetic tweezers. *Rev. Sci. Instrum.* **85**, 123114 (2014).

47. I. De Vlaminck, T. Henighan, M. T. J. Van Loenhout, D. R. Burnham, C. Dekker, Magnetic forces and DNA mechanics in multiplexed magnetic tweezers. *PLOS ONE* **7**, e41432 (2012).

48. M. J. Abraham, T. Murtola, R. Schulz, S. Páll, J. C. Smith, B. Hess, E. Lindahl, GROMACS: High performance molecular simulations through multi-level parallelism from laptops to supercomputers. *SoftwareX* **1-2**, 19–25 (2015).

49. D. H. Paik, V. A. Roskens, T. T. Perkins, Torsionally constrained DNA for single-molecule assays: An efficient, ligation-free method. *Nucleic Acids Res.* **41**, e179 (2013).

50. X. J. Lu, W. K. Olson, 3DNA: A software package for the analysis, rebuilding and visualization of three-dimensional nucleic acid structures. *Nucleic Acids Res.* **31**, 5108–5121 (2003).

51. I. S. Joung, T. E. Cheatham III, Determination of alkali and halide monovalent ion parameters for use in explicitly solvated biomolecular simulations. *J. Phys. Chem. B* **112**, 9020–9041 (2008).

52. U. Essmann, L. Perera, M. L. Berkowitz, T. Darden, H. Lee, L. G. Pedersen, A smooth particle mesh Ewald method. *J. Chem. Phys.* **103**, 8577–8593 (1995).

53. B. Hess, H. Bekker, H. J. C. Berendsen, J. G. E. M. Fraaije, LINCS: A linear constraint solver for molecular simulations. *J. Comput. Chem.* **18**, 1463–1472 (1997).

54. R. W. Hockney, S. P. Goel, J. W. Eastwood, Quiet high-resolution computer models of a plasma. *J. Comput. Phys.* **14**, 148–158 (1974).

55. G. Bussi, D. Donadio, M. Parrinello, Canonical sampling through velocity rescaling. *J. Chem. Phys.* **126**, 014101 (2007).

56. S. Nosé, M. L. Klein, Constant pressure molecular dynamics for molecular systems. *Mol. Phys.* **50**, 1055–1076 (1983).
